# Atypical Hemolytic Uremic Syndrome

**DOI:** 10.1016/j.semnephrol.2013.08.003

**Published:** 2013-11

**Authors:** David Kavanagh, Tim H. Goodship, Anna Richards

**Affiliations:** * The Institute of Genetic Medicine, Newcastle University, Newcastle upon Tyne, UK; † Centre for Inflammation Research, Queen’s Medical Research Institute, University of Edinburgh, Edinburgh, UK

**Keywords:** Complement, eculizumab, factor H, factor I, hemolytic uremic syndrome, membrane cofactor protein, thrombomodulin, transplantation

## Abstract

Hemolytic uremic syndrome (HUS) is a triad of microangiopathic hemolytic anemia, thrombocytopenia, and acute renal failure. The atypical form of HUS is a disease characterized by complement overactivation. Inherited defects in complement genes and acquired autoantibodies against complement regulatory proteins have been described. Incomplete penetrance of mutations in all predisposing genes is reported, suggesting that a precipitating event or trigger is required to unmask the complement regulatory deficiency. The underlying genetic defect predicts the prognosis both in native kidneys and after renal transplantation. The successful trials of the complement inhibitor eculizumab in the treatment of atypical HUS will revolutionize disease management.

The hemolytic uremic syndrome (HUS) is characterized by the triad of thrombocytopenia, microangiopathic hemolytic anemia, and acute renal failure.^1^ The most common form of HUS is secondary to shiga toxin (Stx)-producing bacteria, typically *Escherichia coli* O157:H7. Atypical HUS (aHUS) has been used to classify any HUS not caused by Stx. A variety of precipitating events have been associated with aHUS including infections, drugs, autoimmune conditions, transplants, pregnancy, and metabolic conditions (Table 1). These have frequently been called *secondary aHUS*. With the discovery of the role of complement gene mutations in aHUS, primary aHUS has been used to refer to those cases with documented complement dysregulation. Although a useful aide memoir, these terms do not account for the increasing recognition that patients with an underlying complement risk factor often require a secondary trigger for aHUS to manifest. Classifications that take account of both the genetic background and etiologic trigger are beginning to be introduced.^2^ The best estimate of aHUS incidence is 2 of 10^6^ in a North American population,^3^ although the precise proportion with an underlying complement defect is not known.

**Table 1:** Triggers of aHUS

Trigger	Reference
Non-Stx toxin diarrheal illnesses	51, 94, 95
Norovirus	161, 162
*Campylobacter upsaliensis*	^163^
*Clostridium difficile*	^164^
Respiratory infections	^51^
*Bordetella* pertussis infection	10, 165
*Streptococcus pneumonia*	^166^
*Haemophilus influenzae*	^10^
Other bacterial	
Fusobacterium necrophorum	^167^
Viral illnesses	
Varicella	^168^
Cytomegalovirus	^169^
Influenza H1N1	^170^
Hepatitis A	^171^
Hepatitis C	^172^
Human immunodeficiency virus	^173^
Coxsackie B virus	^174^
Epstein–Barr virus	^175^
Dengue	^176^
HHV6	^177^
Human parvovirus B19	^178^
Parasites	
*Plasmodium falciparum*	^179^
Pregnancy	51, 98, 180
Drugs	
Cisplatin	^181^
Gemcitabine	^182^
Mitomycin	^183^
Clopidogrel	^184^
Quinine	185, 186
Interferon-alfa, -beta	187, 188
Anti–vascular endothelial growth factor	^189^
Campath	^190^
Cyclosporin tacrolimus	^191^
Ciprofloxacin	^192^
Oral contraceptives	193, 194, 195
Illicit drugs (eg, cocaine, heroin, ecstasy)	^196^
Autoimmune	
Anticardiolipin	^197^
C3Nef	^198^
Systemic lupus erythematosus	^199^
Vaccination	
Hepatitis B	^10^
Bone marrow transplantation	^200^
Malignancy (gastric, breast, prostate, lung, colon, ovarian, pancreatic, lymphoma)	^201^
Combined methylmalonic aciduria and homocystinuria	^202^

## Pathology

In acute aHUS, the pathologic picture is of capillary thrombosis. Glomerular capillary wall thickening is seen as a result of endothelial cell swelling and accumulation of flocculent material between the endothelial cell and the basement membrane. Platelet and fibrin thrombi result in occlusion of the glomerular capillaries. Fibrinoid necrosis of the afferent arteriole associated with thrombosis also may be seen. Mesangiolysis occurs early in the disease process and subsequently is replaced by sclerotic changes. Early arterial changes are variable, ranging from only mild endothelial swelling to fibrinoid necrosis with occlusive thrombus formation. Subsequently, there is mucoid intimal hyperplasia with narrowing of the vessel lumen. Deposition of fibrin or fibrinogen in the glomeruli and in the mesangium, as well as within the vessel walls, are seen on immunofluorescence. Complement and immunoglobulin deposits along the capillary loops of glomeruli may be seen.^4^

## The Complement System

**Figure 1:**
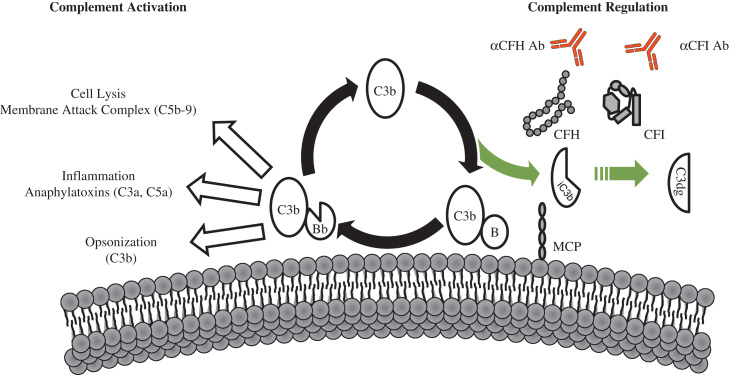
Complement activation and regulation. The AP is a positive amplification loop. C3b interacts with factor B (B), which then is cleaved by factor D to form the AP C3 convertase (C3bBb). This enzyme complex is attached to the target covalently via C3b while Bb is the catalytic serine protease subunit. C3 is the substrate for this convertase, thus creating a powerful feedback loop. Unchecked, this leads to activation of the terminal complement pathway with generation of the effector molecules, the anaphylatoxin C5a, and the membrane attack complex (C5b-9). To protect host cells from bystander damage the AP is down-regulated by complement regulators including CFH, CFI, and MCP. Activating mutations in *C3* and *CFB* and loss-of-function mutations in *CFH*, *CFI*, and *MCP*, in addition to autoantibodies to CFH and CFI, result in overactivation of the AP with resultant aHUS.

The complement system is an ancient defense mechanism that stimulates the inflammatory response and destroys pathogens through opsonization and lysis^5^ (Fig. 1). In addition to protecting the host against invading pathogens, it bridges innate and adaptive immunity and it disposes of immune complexes and injured tissues and cells.^6^

The alternative pathway of complement (AP), which plays a key role in the pathogenesis of aHUS, is continually activated by a tick-over mechanism, and can also be triggered by the classic and lectin pathways.

In the AP, complement C3 undergoes spontaneous hydrolysis, depositing C3b onto the surface of foreign and host cells in the vicinity. On an activating surface such as a bacterium, C3b joins with factor B, which then is cleaved by factor D to form the C3 convertase, C3bBb. The binding of properdin stabilizes this enzyme. This enzyme complex then cleaves more C3 to C3b to initiate a feedback loop. Downstream of this amplification loop, C3b also may join with the C3 convertase to form the C5 convertase. C5 is cleaved to the anaphylatoxin C5a and C5b, which initiates formation of the lytic membrane attack complex (C5b–9) (Fig. 1). To protect host cells from collateral complement damage, many soluble and membrane-associated complement regulatory proteins function to inactivate complement on their surface. It is the imbalance between complement activation and regulation on host cell surfaces that underlies the pathogenesis of aHUS.

## Complement Factor H

**Figure 2:**
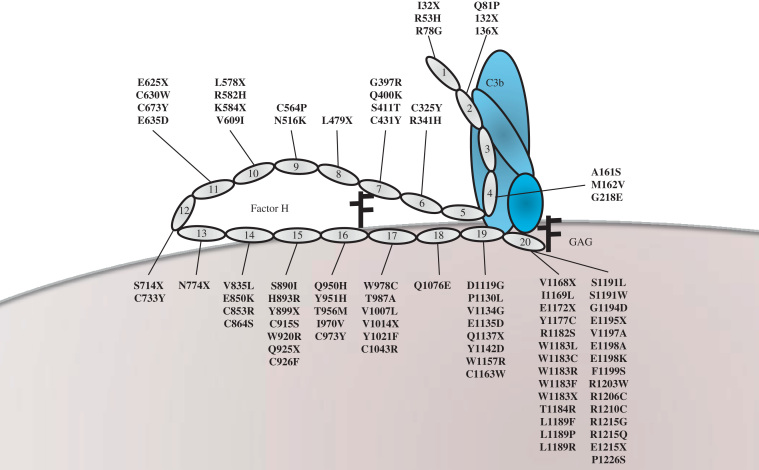
CFH and aHUS-associated mutations. CFH is composed of 20 CCP modules. The N-terminal modules (CCP1-4) bind to C3b and act as a cofactor for the CFI-mediated cleavage to the inactive iC3b. The C-terminal modules (CCP19 and 20) bind to C3b and glycosaminoglycans on host cells to mediate cell surface protection. Genetic variants described in aHUS cluster in CCPs 19 and 20, but can be seen throughout the molecule. Functional analysis of aHUS-associated variants has focused predominantly on the C-terminal variants (Table 3).

Mutations in complement factor H (*CFH*) account for approximately 25% of the genetic predisposition to aHUS (Table 2).^7^, ^8^, ^9^, ^10^, ^11^, ^12^, ^13^, ^14^, ^15^ CFH is the most important fluid-phase regulator of the AP of complement.^16^ CFH is composed of 20 complement control protein modules (CCPs)^17^ (Fig. 2). The four N-terminal CCPs (CCPs 1-4) mediate the complement regulatory functions of the protein by the following: (1) acting as a cofactor for factor I–mediated proteolytic inactivation of C3b, (2) competing with factor B for C3b binding, and (3) accelerating the decay of the C3 convertase into its components.

**Table 2:** Summary Data for Genetic Mutations in aHUS

**Mutations**	CFH*		CFH*	**MCP**		**MCP**	**CFI**		**CFI**	**C3**		**C3**	**THBD**		**THBD**
Reference	^49^		^51^	^49^		^51^	^49^		^51^	^49^		^51^	^49^		^51^
Percentage	27.5		24	9.3		7	8.4		4	8.4		4	0		5
Type I mutation	56%		14%†	91%		88%†	42%		9%†	-		-	N/A		0%†
Type II mutation	44%		86%†	9%		12%†	58%		91%†	-		-	N/A		100%†
Homozygous	1.8%		4%	2.8%		1%	0%		0%	0%		0%	N/A		0%
Heterozygous	25.7%		20%	6.5%		6%	8.4%		4%	8.4%		4%	N/A		5%
	Ped	Ad		Ped	Ad		Ped	Ad		Ped	Ad		Ped	Ad	
Low C3 levels	70%	52%	47%	0%	11%	27%	60%	50%	20%	70%	85%	73%	N/A	N/A	50%
ESRF	52%‡	65%‡	53%§	17%‡	63%‡	6%§	17%‡	83%‡	60%§	43%‡	63%‡	67%§	N/A	N/A	23%§
Death	11%‡	2.5%‡	23%§	0%‡	0%‡	0%§	33%‡	0%‡	0%§	0%‡	0%‡	0%§	N/A	N/A	31%§
Death/ESRF	63%‡	68%‡	77% ^c^	17%‡	63%‡	6%§	50%‡	83%‡	60%§	43%‡	63%‡	67%§	N/A	N/A	54%§

NOTE. A type I mutation results in a quantitative deficiency of the protein, and a type II mutation results in normal levels of a nonfunctional protein.

Abbreviations: Ad, adults; ESRF, end-stage renal failure; N/A, not applicable; Ped, pediatric.

⁎ Includes CFH/CFHR hybrid genes.

‡ Five-year outcome data.

§ Three-year outcome data.

† Estimate was based on mutation, not antigenic levels.

In addition to regulating complement in the fluid phase, CFH also can protect host surfaces by binding to polyanions such as the glycosaminoglycans.^18^ CFH has two glycosaminoglycan binding domains in CCPs 6 to 8 and CCPs 19 and 20,^17^ which have different sulfate specificities resulting in the C-terminal domains (CCP 19 and 20) being predominantly responsible for binding to kidney, and CCPs 6 to 8 being responsible for binding in the eye.^19^ Other recent studies have shown that CFH also binds to the lipid peroxidation product malondialdehyde,^20^ the acute phase proteins, C-reactive protein,^21^, ^22^, ^23^ and pentraxin 3,^24^ as well as necrotic cells.^21^

The majority of mutations in *CFH* are heterozygous, are located in CCPs 19 and 20 (Fig. 2), and do not usually result in a quantitative deficiency of CFH. Structural and functional analysis of the C-terminal mutants has revealed variable consequences on binding to heparin, C3b, and endothelial cells; however, cell surface complement regulation is consistently impaired as measured using sheep erythrocyte lysis assays^25^, ^26^, ^27^ (Table 3). Thus, these C-terminal mutants are predicted to fail to control complement activation at the glomerular endothelium. In keeping with this, renal biopsy data from an aHUS patient with a C-terminal mutant showed reduced CFH binding to renal endothelium compared with wild type.^27^ C-terminal *CFH* mutants also have been shown to have impaired binding to platelets resulting in increased complement activation with consequent platelet activation, aggregation, and release of tissue factor–expressing microparticles.^28^

**Table 3:** Structural and Functional Consequences of *CFH* Mutations in aHUS

**Mutant**	**CCP**	**Structural Changes**	**C3b/d Binding**	**Heparin Binding**	**Cofactor Activity**	**Decay Acceleration Activity**	**Endothelial Cell Binding**	**Hemolysis Assay**	**Reference**
R53H	1	Local	↔	N/A	↓	↓	N/A	↓*	29, 85
R78G	1	ND	↓	N/A	↓	↓	N/A	↓*	^29^
S890I	15	ND	↔	ND	↔	ND	ND	↔†	^30^
V1007L	17	ND	↔	ND	↔	ND	ND	↔†	^30^
D1119G	19	Local	↓‡	↔	N/A	N/A	↔§	↓*	25, 203, 204
Y1142C	19	ND	ND	ND	N/A	N/A	ND	↓†	^26^
W1157R	19	ND	↓	↓	N/A	N/A	ND	ND	^205^
E1172X	20	ND	↓	↓	N/A	N/A	ND	ND	206, 207
R1182S	20	Local	↓	↓	N/A	N/A	ND	↓†, ||	24, 25, 204, 208¶
W1183R	20	Local	↑	↑	N/A	N/A	ND	↓||	25, 204
W1183L	20	Local	↓	↓‡	N/A	N/A	↓‡, §, #	↓||	24, 25, 203, 204, 205, 209¶
T1184R	20	Local	↑‡	↑	N/A	N/A	↑§	↓||	25, 203, 204, 210
L1189R	20	Local	↑	↑	N/A	N/A	↑§	↓†, ||	25, 203, 204, 208
L1189F	20	Local	↑	↑	N/A	N/A	ND	↓†, ||	25, 204, 208
S1191W	20	ND	ND	ND	N/A	N/A	ND	↓†	^208^
S1191L	20	Local	↑‡	↔	N/A	N/A	ND	↓†, ||	25, 31, 211
S1191L/ V1197A	20	Local	↑‡	↔	N/A	N/A	ND	↓†, ||	25, 31, 204, 211
V1197A	20	ND	↓‡	↓‡	N/A	N/A	ND	↓†	31, 204, 205, 208, 209, 211
E1198K	20	ND	ND	ND	N/A	N/A	↓#	↓†	^27^
R1210C	20	Local	↓	↓‡	N/A	N/A	↓#	↓||	25, 204, 205, 207, 208, 209
R1215G	20	Local	↓	↓	N/A	N/A	↓#	↓||	25, 205, 207
R1215Q	20	ND	↔	↓	N/A	N/A	↔§	ND	203, 204
P1226S	20	ND	↓	↓	N/A	N/A	ND	ND	^205^

NOTE. The amino acid numbering refers to the translation start site.

Abbreviations: N/A, not applicable; ND, not done.

⁎ Hemolysis assay using factor H–deficient serum reconstituted with recombinant CFH1-4 in decay acceleration activity (DAA) and cofactor activity (CA) sheep lysis assays.^29^

† Patient serum was used on sheep erythrocytes.^208^

‡ Indicates contradictory results.

§ Endothelial cell binding relates to mGEnC-1^203^ binding.

|| Recombinant proteins competed with full-length *CFH* on human erythrocytes.^25^

¶ Additional experiments have shown R1182S and W1183L have reduced binding to pentraxin 3.^24^

# Endothelial cell binding relates to HUVEC^27^, ^206^, ^207^ binding.

Although *CFH* mutations cluster in the C-terminus, genetic changes are reported throughout the molecule. Mutations in the N-terminal region of *CFH* (CCP 1-4) have been reported and functional analysis has shown that they are defective in their ability to control the AP both in the fluid phase and on cell surfaces.^29^ Sequence variants also have been described in the intervening CCPs of *CFH*, although for many of these changes the functional consequences and importance in pathogenesis remains uncertain. Tortajada et al^30^ examined two of these variants of uncertain significance (VUS) and showed no functional consequences. They went on to show that these variants form part of an infrequent ancient *CFH* haplotype in Caucasians that is not enriched in the aHUS population, highlighting the importance of modeling genetic mutations before ascribing functional significance.

*CFH* resides in the Regulators of Complement Activation (RCA) cluster and the architecture of this region makes it particularly prone to genomic rearrangements. The gene for *CFH* is in close proximity to the genes encoding the five factor H–related proteins that are thought to have arisen from several large genomic duplications and thus have a very high degree of sequence identity to *CFH*.

This homology predisposes to gene conversions and genomic rearrangements through nonallelic homologous recombination and microhomology-mediated end joining. The *CFH* mutations S1191L, V1197A, and combined S1191L/V1197A arose through gene conversion between *CFHR1* and *CFH*.^31^ A hybrid (fusion) gene comprising the 21 N-terminal exons of *CFH* and the 2 C-terminal exons of *CFHR1* was shown to have arisen through nonallelic homologous recombination and resulted in aHUS.^32^ More recently, a hybrid gene consisting of the 22 N-terminal exons of *CFH* and the 5 C-terminal domains of *CFHR3* arising through microhomology-mediated end joining was reported in aHUS.^33^ As with C-terminal point mutations in *CFH*, these hybrid genes also result in loss of cell-surface complement regulation.

A transgenic mouse lacking the C-terminal domains of CFH (Cfh^-/-^Δ16–20) was generated to provide an in vivo model of aHUS.^34^ These mice spontaneously developed aHUS, confirming the importance of local endothelial cell complement regulation^35^ in vivo. Goicoechea de Jorge et al^36^ also have crossed the Cfh^-/-^Δ16–20 mouse with a C5-deficient mouse to investigate the role of C5 activation in the pathogenesis of aHUS. These C5^-/-^ CFH^-/-^Δ16–20 mice do not develop aHUS, suggesting a critical role downstream of C3b generation in aHUS.

## Complement Factor I

**Figure 3:**
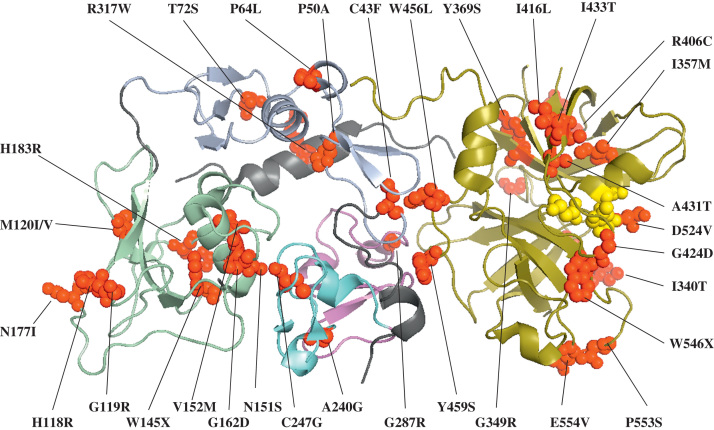
Location of aHUS-associated mutations within the crystal structure of factor I (protein database identification code: 2XRC).^160^ Factor I is a heterodimer consisting of a noncatalytic heavy chain linked by a disulfide bond to a catalytic light chain. The domain structure of CFI is shown with the heavy chain comprising the FIMAC domain, light blue; SRCR domain, pale green; LDLr1, cyan; and LDLr2, magenta; and the light chain or serine protease domain, deep olive. aHUS-associated genetic variants are shown as red spheres. Yellow spheres mark the catalytic triad of the serine protease domain. Functional analysis of aHUS-associated *CFI* variants are described in Table 4.

Mutations in complement factor I (*CFI*) account for between 5% and 10% of aHUS (Table 2, Table 4).^11^, ^37^, ^38^, ^39^, ^40^, ^41^, ^42^, ^43^, ^44^ CFI is a serum serine protease that functions as a critical mediator of complement regulation by cleaving C3b and C4b in the presence of its cofactors (CFH for C3b; C4b binding protein for C4b; membrane cofactor protein [MCP] and complement receptor 1 for both). It is synthesized predominantly by the liver. The *CFI* mutations described in aHUS are all heterozygous. These mutations cluster in the serine protease domain (Fig. 3 and Table 4).

**Table 4:** Mutations in CFI Reported in aHUS and Functional Consequences

**Mutation**	**Domain**	**Serum CFI Level**	**Recombinant Secretion**	**Fluid Phase C3 Cofactor Activity**	**Fluid Phase C4 Cofactor Activity**	**Cell Surface Activity**	**Reference**
C43F	FIMAC	↓	↓	N/A	N/A	N/A	41, 49, 93
P50A	FIMAC	↓	↓*	↓†	↓‡	↓	41, 49, 93
P64L	FIMAC	N/D	N/D	N/D	N/D	N/D	^11^
T72S	FIMAC	N/D	N/D	N/D	N/D	N/D	^51^
H118R	CD5	↔	N/D	N/D	N/D	N/D	49, 93
G119R	CD5	↔	N/D	N/D	N/D	N/D	11, 49, 93
M138I	CD5	↔	↔	↔	↔	N/D	^39^
M138V	CD5	↔	↓	↔	↔	↑	41, 93
W145X	CD5	↓	↓	N/A	N/A	N/A	38, 41
N151S	CD5	↓	↓	N/A	N/A	N/A	41, 49, 93
V152M	CD5	N/D	N/D	N/D	N/D	N/D	^43^
G162D	CD5	↓	N/A	N/A	N/A	N/A	44, 147
N177I	CD5	N/D	N/D	N/D	N/D	N/D	^46^
H183R	CD5	↔	↔	↔	↔	↔	11, 41, 49, 212
A240G	LDLr1	↔	↓	↔	↔	↓	40, 41, 51
C247G	LDLr1	N/D	N/D	N/D	N/D	N/D	^46^
C249G	LDLr1	↓	N/D	N/A	N/A	N/A	^92^
G261D	LDLr2	↔	↔	↔	↔	↔	39, 42, 49, 51
G287R	LDLr2	N/D	N/D	N/D	N/D	N/D	^11^
c.784delA	LDLr2	↓	↓	N/A	N/A	N/A	^93^
c.893delC	LDLr2	↓	↓	N/A	N/A	N/A	38, 41
I306S	LDLr2	↔	N/D	N/D	N/D	N/D	^49^
R317W	SP link	↔	↓	↓*	↓*	↔	39, 40, 41, 51
I340T	SP link	↔	↔	↓	↓	N/D	39, 213
G342E	SP	↔	N/D	N/D	N/D	N/D	^49^
I344V	SP	↔	N/D	N/D	N/D	N/D	^49^
G349R	SP	N/D	N/D	N/D	N/D	N/D	^51^
I357M	SP	↔	N/D	N/D	N/D	N/D	49, 51
Y369S	SP	N/D	N/D	N/D	N/D	N/D	^214^
W399R	SP	N/D	N/D	N/D	N/D	N/D	^51^
D403N	SP	↔	N/D	N/D	N/D	N/D	49, 93
R406C	SP	↔	N/D	N/D	N/D	N/D	^67^
I416L	SP	↓	↓	N/A	N/A	N/A	49, 93, 94
G424D	SP	↔	N/D	N/D	N/D	N/D	^93^
A431T	SP	↓	↓	N/A	N/A	N/A	49, 93
I433T	SP	↔	N/D	N/D	N/D	N/D	93, 94
K441R	SP	↔	N/D	N/D	N/D	N/D	^215^
W456L	SP	↓	↓	N/A	N/A	N/A	49, 93
Y459S	SP	↔	N/D	N/D	N/D	N/D	37, 49
R474X	SP	↓	↓	N/A	N/A	N/A	37, 41, 43, 49
c.1446-1450del TTCAC	SP	↔	↓	N/A	N/A	N/A	39, 40, 41
D519N	SP	N/D	↔	↓	↓	↓	39, 40, 41
K522T	SP	N/D	N/D	N/D	N/D	N/D	^11^
D524V	SP	↔	↔	↓*	↓*	↓	37, 39, 49, 93
c.1610ins AT	SP	↓	↓	N/A	N/A	N/A	41, 80
W546X	SP	↓	↓	N/A	N/A	N/A	37, 41
E554V	SP	N/D	N/D	N/D	N/D	N/D	^51^
P553S	SP	↔	N/D	N/D	N/D	N/D	49, 93

NOTE. The amino acid numbering refers to the translation start site. The previously reported rare genetic variant IVS12+5G>T was not included because it is not enriched in an aHUS population.

Abbreviations: N/A, not applicable; ND, not done; FIMAC, factor-I membrane attack complex domain; LDLr, low-density lipoprotein receptor domains; SP, serine protease domain.

⁎ Contradictory result.

† Only seen using CFH as a cofactor.

‡ Only seen using C4b binding protein.

Functional analysis has been performed for a number of mutants and shows a loss of both alternative and classic pathway regulatory activity in the fluid phase and on cell surfaces (Table 4).^39^, ^41^, ^42^ As seen in *CFH*, several VUS have been described in *CFI* in which analysis has failed to show any alteration in secretion or function (eg, G261D). Such variants may not be involved in the pathogenesis of disease.

## Membrane Cofactor Protein

Mutations in *MCP* (CD46) are found in approximately 10% of patients with aHUS^11^, ^40^, ^43^, ^44^, ^45^, ^46^, ^47^ (Table 2). MCP is a surface-bound complement regulatory protein that acts as a cofactor for the CFI-mediated cleavage of C3b and C4b that are deposited on host cells.^48^

**Figure 4:**
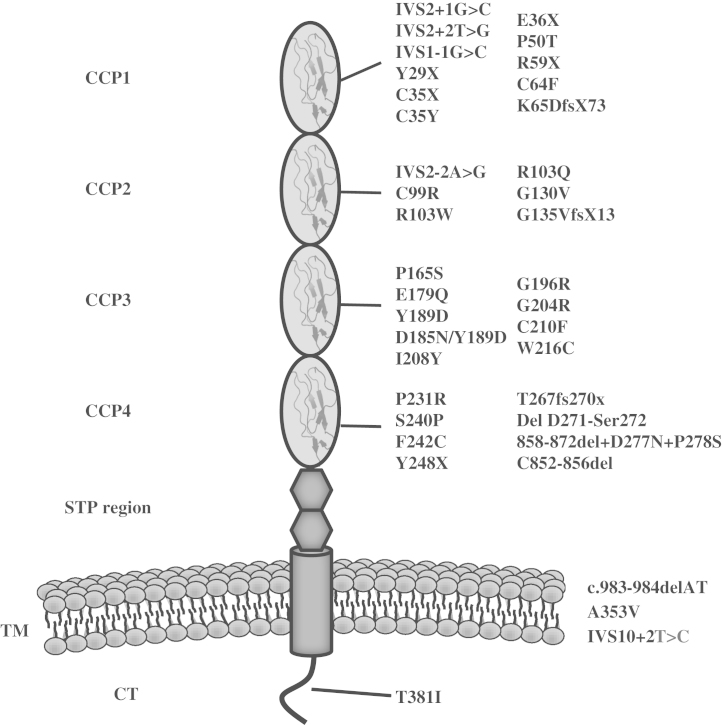
Mutations in MCP associated with aHUS. MCP is a transmembrane glycoprotein. It consists of 4 CCPs. Following the CCPs is an alternatively spliced region, rich in serine, threonine, and proline (STP region). The STP region is followed by a group of 12 amino acids of unknown function, a hydrophobic domain, a charged transmembrane anchor, and the alternatively spliced cytoplasmic tail (CT). Mutations associated with aHUS are clustered in the four extracellular CCPs of the molecule. Functional analysis of aHUS-associated *MCP* variants are described in Table 5.

The majority of mutations described in aHUS are found in the extracellular four CCP domains that are responsible for C3b and C4b binding (Table 5 and Fig. 4). Most *MCP* mutations described to date have resulted in a quantitative defect in MCP (~75%). The remaining mutations have been shown to result in a secreted, nonfunctional protein (Table 5).

**Table 5:** Mutations in MCP Reported in aHUS and Functional Consequences

**Mutation**	**Domain**	**Expression**	**C3b Binding**	**C3 Cofactor Activity**	**C4b Binding**	**C4b Cofactor Activity**	**Reference**
IVS2+1G>C	1	↓	N/A	N/A	N/A	N/A	48, 80
IVS2+2T>G	1	↓	N/A	N/A	N/A	N/A	11, 43, 44, 47, 48
IVS1-1G>C	1	↓	N/A	N/A	N/A	N/A	40, 48
Y29X	1	N/D	N/D	N/D	N/D	N/D	^46^
C35X	1	↓	N/A	N/A	N/A	N/A	^51^
C35Y	1	↓	N/A	N/A	N/A	N/A	40, 48, 51
E36X	1	↓	N/A	N/A	N/A	N/A	^49^
P50T	1	N/D	N/D	N/D	N/D	N/D	^51^
R59X	1	↓	N/A	N/A	N/A	N/A	40, 44, 47, 51
C64F	1	↓	N/A	N/A	N/A	N/A	^168^
K65DfsX73	1	N/D	N/D	N/D	N/D	N/D	^46^
IVS2-2A>G	2	↓	N/A	N/A	N/A	N/A	11, 40, 47, 48, 107, 170
C99R	2	↓	N/A	N/A	N/A	N/A	40, 48
R103W	2	↔	↔	↔*	↔	↔	48, 216
R103Q	2	N/D	N/D	N/D	N/D	N/D	^46^
G130V	2	N/D	N/D	N/D	N/D	N/D	^46^
G135VfsX13	2	N/D	N/D	N/D	N/D	N/D	^44^
P165S	3	↓	N/A	N/A	N/A	N/A	46, 48, 80
E179Q	3	↑	↔	↔	↔	↓	47, 48
Y189D	3	↓	N/A	N/A	N/A	N/A	9, 11, 47, 48, 49, 51
D185N/Y189D	3	↓	N/A	N/A	N/A	N/A	47, 48
I208Y	3	N/A	N/A	N/A	N/A	N/A	^46^
G196R	3	↓	N/A	N/A	N/A	N/A	^48^
G204R	3	N/D	N/D	N/D	N/D	N/D	^46^
C210F	3	↓	N/A	N/A	N/A	N/A	46, 92
W216C	3	N/D	N/D	N/D	N/D	N/D	^11^
P231R	4	N/D	N/D	N/D	N/D	N/D	^11^
S240P	4	↔	↓	↓	↔	↔	45, 48
F242C	4	↔	↓	↓	↓	↓	11, 46, 48, 51
Y248X	4	↓	N/A	N/A	N/A	N/A	47, 48
T267fs270x	4	↓	N/A	N/A	N/A	N/A	48, 51, 217
Del D271-Ser272	4	↓	N/A	N/A	N/A	N/A	43, 45, 63
858-872del+D277N+P278S	4	↓	N/A	N/A	N/A	N/A	40, 48
C852-856del	4	↓	N/A	N/A	N/A	N/A	48, 80
c.983-984delAT	TM	N/D	N/A	N/A	N/A	N/A	^11^
A353V	TM	↔	↔*	↔	↔	↔	40, 48, 216
IVS10+2T>C	TM	↓	N/A	N/A	N/A	N/A	49, 218
T381I	CT	N/D	N/D	N/D	N/D	N/D	^46^

NOTE. The amino acid numbering refers to the translation start site.

Abbreviations: CT, cytoplasmic tail; N/A, not applicable; N/D, not done; TM, transmembrane.

⁎ Inability to control complement was detected on cell surface assays only.

## Activating Mutations in Complement Components

Mutations have been described more recently in the complement components *C3* and complement factor B (*CFB*). C3 is cleaved to form the anaphylatoxins C3a and C3b, which are highly reactive, and can bind to cell surfaces via their reactive thioester. C3b then can interact with CFB in the presence of factor D to form the AP C3 convertase (C3bBb), which cleaves further C3, introducing a positive-feedback loop.

**Figure 5:**
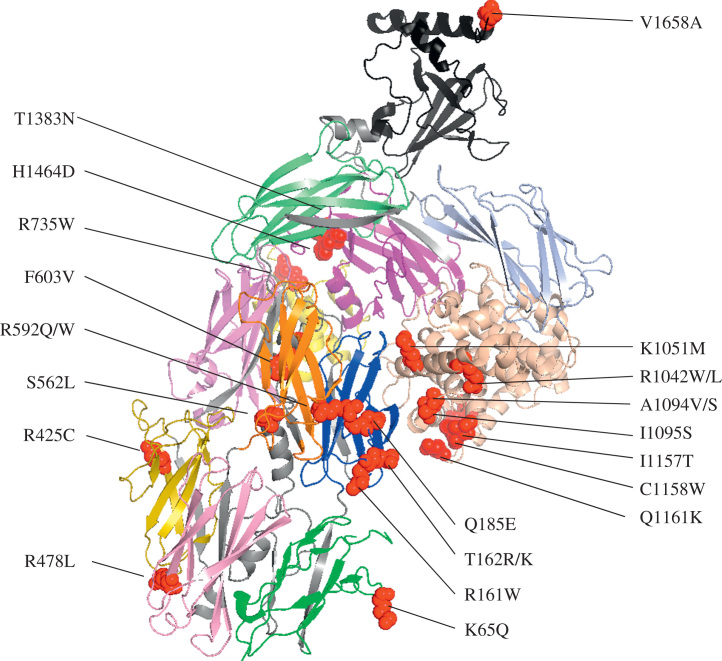
Location of aHUS-associated mutations within the crystal structure of C3 (protein database identification code: 2A73). The structure of C3 is represented with the domains highlighted: MG1, green; MG2, blue; MG3, violet; MG4, olive; MG5, pink; MG6, orange; ANA, yellow; α’NT, grey; MG7, lime; CUB, light blue; TED, wheat; MG8, purple; and C345C, black. Genetic variants (red spheres) cluster around the MG2 and TED domains. Functional analysis of C3 mutations in aHUS has been performed in only a few cases (Table 6).

Mutations in *C3* have been reported in several cohorts of aHUS patients^9^, ^10^, ^11^, ^49^, ^50^, ^51^, ^52^, ^53^, ^54^ at a frequency of 2% to 10% (Table 2). Initial functional analysis showed that MCP was unable to bind to mutant C3, preventing its cleavage to iC3b.^50^ Two C3 mutations have been described that resulted in decreased secretion and their role in pathogenesis remains uncertain. More recently, two mutations in C3 have been reported that bind to CFB with higher affinity, resulting in increased C3 convertase formation.^52^, ^55^ These mutations result in increased complement activation on platelets^52^ and glomerular endothelium^55^ (Fig. 5 and Table 6).

**Table 6:** Mutations in C3 Reported in aHUS Documenting Functional Analysis

**Mutation**	**Domain**	**Expressed**	**MCP Binding**	**CA Activity**	**Factor H Binding**	**Factor B Binding**	**C3 Convertase Formation**	**Reference**
K65Q	MG1	Yes	N/D	N/D	↓	N/D	N/D	49, 219
R161W	MG2	Yes	↓	↓	↔ *	↑	↑	10, 49, 54
T162R	MG2	N/D	N/D	N/D	N/D	N/D	N/D	^51^
T162K	MG2	N/D	N/D	N/D	N/D	N/D	N/D	^51^
Q185E	MG2	N/D	N/D	N/D	N/D	N/D	N/D	^51^
R425C	MG4	N/D	N/D	N/D	N/D	N/D	N/D	^9^
R478L	MG5	N/D	N/D	N/D	N/D	N/D	N/D	^51^
S562L	MG6b	N/D	N/D	N/D	N/D	N/D	N/D	^9^
R592Q	MG6b	Yes	↓	↓	↔†	↔	N/D	50, 53
R592W	MG6b	Yes	↓	↓	↔†	↔	N/D	50, 51
F603V	MG6b	N/D	N/D	N/D	N/D	N/D	N/D	^11^
R735W	ANA	Yes	↔	↔	↔	↔	N/D	^50^
V762I	α’NT	N/D	N/D	N/D	N/D	N/D	N/D	^51^
Y854X	MG7	No	N/A	N/A	N/A	N/D	N/D	49, 50
R1042W	TED	N/D	N/D	N/D	N/D	N/D	N/D	^51^
R1042L	TED	N/D	N/D	N/D	N/D	N/D	N/D	^11^
K1051M	TED	N/D	N/D	N/D	N/D	N/D	N/D	^51^
A1094V	TED	Yes	↓	↓	↓	↔	N/D	^50^
A1094S	TED	N/D	N/D	N/D	N/D	N/D	N/D	^49^
I1095S	TED	N/D	N/D	N/D	N/D	N/D	N/D	^49^
P1114L	TED	N/D	N/D	N/D	N/D	N/D	N/D	^49^
D1115N	TED	Yes	↓	↓	↓	↔	N/D	50, 51
I1157T	TED	N/D	N/D	N/D	N/D	N/D	N/D	9, 11, 51
C1158W	TED	No	N/A	N/A	N/A	N/D	N/D	^50^
Q1161K	TED	Yes	↓	↓	↓	↔	N/D	^50^
T1383N	MG8	N/D	N/D	N/D	N/D	N/D	N/D	^51^
H1464D	MG8	Yes	↔	↔	↔	↔	N/D	^49^
V1658A	C345C	Yes	N/D	N/D	↔	↑	↑	^52^

NOTE. The amino acid numbering refers to the translation start site.

Abbreviations: N/A, not applicable; N/D, not done; MG, macroglobulin domains; TED, thioester-containing domain; α’NT, N-terminal region of the cleaved α-chain; ANA, anaphylatoxin domain.

⁎ Reported elsewhere to have decreased Factor H binding.^219^

† Reduced but nonsignificant.

Gain-of-function mutations also have been reported in *CFB* although these appear to be rare.^9^, ^10^, ^51^, ^56^, ^57^, ^58^ Goicoechea de Jorge et al^56^ described two mechanisms through which these separate mutations led to increased complement activation. One mutant (F286L) showed enhanced formation of the C3bB proenzyme that resulted in a more active enzyme in vivo. The other mutant (K323E) formed a C3bBb enzyme more resistant to decay by the complement regulators decay accelerating factor (CD55) and CFH. This also caused increased enzyme activity.^56^ Subsequent analysis of two further mutants located in the von Willebrand type A domain (D279G and K350N) showed increased convertase formation and resistance to CFH dissociation, ultimately resulting in increased complement deposition on endothelial cells.^55^

## Thrombomodulin

Thrombomodulin (*THBD*) facilitates the activation of protein C by thrombin^59^ and enhances thrombin-mediated activation of plasma procarboxypeptidase B (CPB2), an inhibitor of fibrinolysis that also inactivates complement-derived anaphylatoxins C3a and C5a. It has also been shown to down-regulate the AP by accelerating CFI-mediated inactivation of C3b in the presence of cofactors.^60^ Mutations in *THBD* recently were described in aHUS. The variations reported were heterozygous missense mutations, with no mutations resulting in a loss of secretion. These mutations resulted in a loss of cofactor activity.^60^ Maga et al^11^ also have reported *THBD* genetic variants in aHUS, although they suggested that at least one of the previously reported mutations was a polymorphism present in 3% of the population and that several *THBD* variants were present with an additional mutation. No isolated *THBD* mutations were described in a large French cohort of patients (n = 214), although a few individuals carried *THBD* genetic variants in addition to a mutation in a known complement gene.^49^ Several large cohorts of aHUS have yet to report on the frequency of genetic variants in *THBD* in aHUS.

## Other Genetic Variants

Genetic screening of complement factor H–related 5 protein (*CFHR5*) in 3 separate cohorts of aHUS patients was performed.^11^, ^61^, ^62^ Monteferrante et al^62^ did not detect any causative mutations in an Italian cohort. Westra et al^61^ reported 3 VUS in *CFHR5* in a panel of 65 aHUS patients whereas Maga et al^11^ reported 3 VUS in aHUS (n = 144) patients, 2 of whom carried an additional known mutation. No mutation was seen to segregate in a large pedigree with the reported cases being sporadic. The current limited understanding of the functional role of CFHR5 further adds to the uncertainty of its role in aHUS pathogenesis and replication studies are required.

A functionally significant mutation (Q433P) in the membrane attack complex regulator, *clusterin*, has been reported in a family with aHUS.^63^ The affected individual also carried a functionally significant mutation in *MCP*; therefore, it is unclear whether mutations in *clusterin* are sufficient alone to cause aHUS.

## Factor H Autoantibodies

In addition to the genetic abnormalities described in aHUS, autoantibodies to CFH also have been linked to disease in 4% to 14% of aHUS patients (Table 7).^10^, ^51^, ^64^, ^65^, ^66^, ^67^, ^68^ In a pediatric-only cohort, this figure was reported to be as high at 25%.^69^

**Table 7:** CFH-Autoantibody Associated aHUS

	**Dragon-Durey et al**^**65**^	**Noris et al**^**51**^	**Moore et al**^**67**^	**Abarrategui-Garrido et al**^**64**^	**Jozsi et al**^**66**^	**Geerdink et al**^**10**^
Percentage aHUS	7^49^	4	9	4.6	11	13
Relapse	59%	37.5%	23%	N/A	N/A	60%
Children	84%	75%	100%	100%	100%	100%
Adult	16%	25%	0%	0%	0%	0%
Low C3 levels	58%	43%	23%			
Long-term follow-up evaluation						
ESRF	27%	63%	46%	28.5%	N/A	0%
Death	9.5%	0%	0%	14%	N/A	16.6%
Death/ESRF	36.5%	63%	46%	42.5%	N/A	16.6%

Abbreviations: ESRF, end-stage renal failure; N/A, not applicable.

CFH autoantibodies in aHUS are strongly associated with an 80-kb–long genomic homozygous deletion of *CFHR*1 and *CFHR*3.^10^, ^65^, ^66^, ^70^ More recent analysis also has shown that some aHUS patients with *CFHR*1 deficiency resulting from point mutations in *CFHR*1^71^ or from a deletion incorporating *CFHR*1 and *CFHR*4^71^, ^72^ have developed anti-CFH antibody (Ab). This may suggest that deficiency of *CFHR1* is the predominant predisposing factor in the development of autoantibodies. Despite this, deficiency of *CFHR1* is not a prerequisite for formation of autoantibodies because several aHUS patients have been reported with high titers of Ab with no evidence of deficiency of *CFHR1* or *CFHR3*.^10^, ^67^, ^73^, ^74^ In many cases, aHUS patients with anti-CFH Ab also carried functionally significant mutations in other complement genes.^67^

The anti-CFH antibodies reported to date have been predominantly of the IgG class, although a few IgA anti-CFH Ab have been described.^74^ Mapping of the epitopes initially suggested that the anti-CFH Ab bound predominantly to the C-terminus,^66^, ^67^, ^75^, ^76^ however, recently it was reported that the response was polyclonal to multiple epitopes throughout CFH.^77^ There was also cross-reactivity with CFHR1^67^, ^74^, ^77^ and CFHR2,^77^ but it was not reported for CFHR3 or CFHR4A.^74^

Several studies have shown various functional consequences of anti-CFH Abs. The antibodies have been shown to reduce binding to C3b^75^, ^77^ and other C3 fragments.^77^ They perturb CFH-mediated cell surface protection^75^, ^77^ and in some individuals the autoantibodies also impair cofactor activity^77^ or decay accelerating activity.^76^ In keeping with this, low C3 levels frequently are seen in the autoimmune form (Table 7).

It also has been shown that CFH autoantibodies form immune complexes in the serum,^74^, ^77^ which may explain the low CFH levels seen in 28% of the cases.^77^ In addition, these immune complexes correlated with disease activity.^77^ In summary, these functional studies suggest a pathogenic role for CFH autoantibodies in aHUS.

## Factor I Autoantibodies

Autoantibodies to CFI also have been described in aHUS but are much rarer than anti-CFH Abs (0%-2%).^68^, ^78^ Unlike anti-CFH Abs they are not associated with a deletion of *CFHR1* and 3. Anti-CFI Abs were seen to form immune complexes in serum, however, functional analysis showed only a minor effect in fluid phase cofactor activity.^78^ The co-existence of functionally significant mutants in the majority of patients, added to the lack of correlation of anti-CFI Ab titer and disease activity, raise the possibility that they are an epiphenomenon rather than a direct cause of disease. Large replication studies will be needed to confirm this initial observation.

## Genetic Susceptibility Factors

A number of single-nucleotide polymorphisms (SNPs) in *CFH* have been shown to be associated with aHUS in several studies.^34^, ^64^, ^79^, ^80^, ^81^, ^82^ A haplotype in *CFH* (*CFH*-H3; tgtgt) composed of these SNPs increases this risk of aHUS two- to four-fold.^34^, ^49^ This haplotype contains a SNP in the region of *CFH* responsible for cofactor activity. Functional analysis has shown that the risk variant, *CFH*-Val_62_, has a subtle decrease in cofactor activity compared with the protective variant,^29^, ^83^, ^84^ in keeping with the minor structural differences between these SNPs.^85^

A haplotype block in *MCP* (*MCP*ggaac) comprising 2 SNPs in the promoter region has been associated with a two- to three-fold increased risk of aHUS.^49^, ^80^, ^81^ Some of these studies have suggested that this risk occurs exclusively in those patients already carrying complement mutations.^80^, ^82^ Reporter gene assays have suggested that this haplotype reduces transcriptional activity by 25%,^80^ however, this did not correlate with MCP cell surface expression in vivo.^80^, ^86^, ^87^ Further experiments on human umbilical vein endothelial cells (HUVECs) with risk and protective *MCP* haplotypes showed equal MCP expression whether in the resting state, after cytokine treatment, or free heme treatment. Likewise, no increase in complement deposition could be shown on HUVECs bearing the *MCP* risk haplotype.^87^

A SNP in C4b binding protein (R240H) was associated with aHUS in cohorts from the United Kingdom and France. C4b binding protein is the predominant classic pathway fluid phase regulator but also has weak AP regulatory activity. Functional analysis showed inefficient AP regulation compared with wild type.^88^ In a replication study in a Spanish cohort this association could not be confirmed.^89^

A *CFHR1* polymorphism (CFHR1*B) resulting from a gene conversion between *CFH* and *CFHR1* is associated strongly with aHUS in the homozygous state,^64^ an association replicated by Fremeaux-Bacchi et al.^49^ It has been suggested that because the CFHR1*B is identical to CFH in CCP18 that it may compete with CFH at the glomerular endothelium, thus impairing complement regulation.

In a study examining SNPs in 47 complement genes in two separate cohorts, SNPs in *CFHR2* and *CFHR4* were also associated with aHUS. In this study there were no reproducible associations between SNPs and aHUS outside the RCA cluster.^82^

## Incomplete Penetrance

Incomplete penetrance has been reported for all the genes associated with aHUS. Penetrance has been reported at around 50% for individuals carrying *CFH*, *CFI*, *MCP*, and *CFB* mutations,^40^, ^90^ and slightly lower for *C3* mutations, albeit with small numbers.^53^ In index cases, the age-related penetrance is significantly higher than their mutation-positive relatives regardless of gene.^91^ This suggests that the penetrance is altered by other genetic and environmental modifiers.

Patients have been reported with mutations in more than one complement gene^11^, ^86^, ^92^, ^93^, ^94^ or mutations in one complement gene in addition to autoantibodies to complement components.^67^, ^78^ In a study of 795 aHUS patients the European Working Party on Complement Genetics showed that at least 3.4% of aHUS cases will have more than one mutation. Eight percent to 10% of patients with mutations in *CFH*, *C3*, or *CFB* had combined mutations whereas 25% of patients with mutations in *CFI* or *MCP* had combined mutations.^46^ The penetrance increased as the number of mutations in a patient increased.^46^ As described, haplotypes and SNPs act together with mutations and inhibitory autoantibodies to increase the penetrance of disease.

Even in the situation in which a patient has multiple genetic risk factors, disease may not manifest until middle age, suggesting a triggering stimulus is required for disease to manifest. In such cases, it is likely that aHUS results from an otherwise innocuous stimulus that triggers the AP and sets off a self-amplifying cycle that cannot be controlled adequately in susceptible individuals.

## Triggering Events

Historically, many agents have been linked to aHUS (Table 1). Although many of these associated conditions are common, aHUS is rare, suggesting an underlying predisposition.

Recent analysis of cohorts of aHUS patients with complement mutations have identified upper respiratory tract infections, fevers, pregnancy, drugs, and non *E coli* diarrheal illnesses as potential triggers.^40^, ^95^, ^96^, ^97^ Non-Stx diarrhea preceded aHUS in 23% of a French cohort^94^ and in 28% of an Italian cohort.^51^ Infectious events, mainly upper respiratory tract infections or diarrhea/gastroenteritis, trigger onset of aHUS in at least half of patients.^49^, ^51^ Likewise, in pregnancy-associated aHUS, Fakhouri et al^98^ showed that 86% of patients for whom this was a trigger carried a complement gene mutation. Drugs also may unmask latent complement defects resulting in aHUS.^40^, ^99^

## Complement Screening in aHUS

Once the initial diagnosis of a thrombotic microangiopathy (TMA) has been made, the initial management involves differentiating between Stx-HUS, thrombotic thrombocytopenic purpura, and aHUS (for a diagnostic algorithm see the article by Loirat and Fremeaux-Bacchi^100^). Rapid exclusion by analysis of a disintegrin and metalloproteinase with a thrombospondin type 1 motif, member 13 (ADAMTS13) activity and microbiological analysis for Stx-producing *E coli* can lead to a diagnosis of aHUS. After exclusion of Stx-HUS and thrombotic thrombocytopenic purpura, precipitating events and the underlying genetic defects predisposing to aHUS should be sought (Table 8).

**Table 8:** Evaluation of Thrombotic Microangiopathies

Hematologic	Thrombocytopenia
	Microangiopathic hemolytic anemia (typically Coombs-negative)
Biochemical	Increased lactate dehydrogenase
	Increased creatinine
Urinary	Proteinuria
	Hematuria
Shiga-toxin *E. Coli* infection	Stool/rectal swab culture
	Polymerase chain reaction STX
	Anti-lipopolysaccharide antibodies
Thrombotic thrombocytopenic purpura	ADAMTS13 activity
Complement analysis	C3, C4
	CFH, CFI, C3Nef
	MCP fluorescence-activated cell sorter analysis
	Anti–factor H and I autoantibodies
	Genetic analysis CFH, CFI, CFB, C3 MCP (including copy number variation)
*S pneumoniae*	Culture
	Positive T-antigen
Pregnancy	Pregnancy test
Virology	Human immunodeficiency virus serology
	H1N1 serology
	Cytomegalovirus polymerase chain reaction
	Epstein-Barr virus polymerase chain reaction
Autoimmune diseases	ANA
	Anti–double-stranded DNA
	Antiphospholipid Ab
Metabolic	Plasma amino acid chromatography
	Urine organic acid chromatography
	Genetic analysis

Complement analysis in cases of aHUS should include serum levels of C3, C4, CFH, and CFI before plasma exchange. Low C3 levels are seen commonly in patients with mutations in *CFH*, *CFI*, *MCP*, *C3*, and *CFB* and may point to a complement-mediated process, however, normal C3 levels do not exclude the presence of a mutation in, or autoantibodies against, the complement system (Table 2).^101^ Fluorescence-activated cell sorter analysis of peripheral blood mononuclear cells provides a quick and relatively inexpensive screening option for *MCP* mutations, although genetic analysis still is required to detect all changes.

Genetic screening in aHUS is challenging because most of the disease-associated mutations are individually rare. In the case of nonsense mutations, large gene rearrangements, and frame shift mutations, the functional consequences are certain. In aHUS, missense mutations or splice-site variations in complement genes comprise a high proportion of the variants found and the changes may be of uncertain biologic or clinical relevance. These VUS pose a challenge when reporting the genetic results.^102^ Although predictions can be made as to the likely significance of a VUS, ultimately, functional assays are required to assess the importance of a variant. Such analysis often has led to the reclassification of a previously described mutation as an irrelevant polymorphism.^30^ Care always should be taken when interpreting these VUS and screening laboratories should revisit past genetic data in light of new evidence. In addition to direct DNA sequencing, the not-insubstantial number of gene conversions and genomic rearrangements found in aHUS makes copy number analysis essential in aHUS screening (see www.genetests.org for national screening laboratories).

## Prognosis

The overall prognosis for patients with aHUS has been poor. Initial mortality has been reported to be higher in children (6.7% versus 0.8% at 1 year),^49^ although adults progress to end-stage renal disease (ESRD) more frequently at initial presentation (46% versus 16%).^49^ At 3 to 5 years after onset, 36% to 48%^49^, ^51^ of children and 64% to 67%^49^, ^51^ of adults had died or reached ESRD.

Prognosis varies with genotype, with *MCP* mutations carrying the best prognosis,^49^, ^51^, ^94^ although in one study this was true only if the first presentation of aHUS occurred in childhood^49^ (Table 2). No patient with an *MCP* mutation from either the French^49^ or Italian cohorts^51^ died at first episode and none of the children and only 25% of adults with an *MCP* mutation developed ESRD at first episode. At 3 years only 6% of all patients with *MCP* mutations^51^ had developed ESRD and by 5 years only 35% had developed ESRD.^49^

Individuals with mutations in *CFH*, *CFI*, or *C3* all had poor outcomes. In patients with a *CFH* mutation the initial mortality rate was 30% in children and 4% in adults,^49^ and evolution to ESRD at first episode in survivors was 19% to 33% in children and 48% in adults.^49^, ^51^ At 3 to 5 years of follow-up evaluation, up to 77% of patients with *CFH* mutations had developed ESRD or had died. Only 30% to 40% of individuals with *CFI* and *C3* mutations will be alive with native kidney function at 3 to 5 years.^49^, ^51^ The prognosis of aHUS with *CFB* mutations also is poor.^51^, ^55^, ^56^

A proportion of patients will have combined mutations. In patients with either *CFH* or *CFI* mutations, the presence of mutations in other genes did not modify prognosis. In contrast, the prognosis for individuals with an *MCP* mutation was worse if they also carried an additional mutation.^46^

In those patients with CFH autoantibodies, 36.5% to 63% die or reach ESRD in the long term^49^, ^51^, ^64^, ^65^, ^66^, ^67^ (Table 7).

## Extrarenal Manifestations

Extrarenal manifestations are reported in only 10% to 20% of patients with aHUS. The most frequently reported symptoms (~10%) are neurologic, with symptoms ranging from irritability to coma. It is unclear how many of these symptoms are the direct result of a cerebral TMA, with severe hypertension and posterior reversible encephalopathy syndrome possible differential diagnoses that may be differentiated on magnetic resonance imaging.^103^ Many case reports of other organ involvement occurring concurrently with aHUS have been described (Table 9), but in the majority, definitive biopsy evidence of TMA in the organ was lacking. Extrarenal manifestations have been reported more commonly in CFH-autoantibody–mediated disease, with 23.5% having seizures and 23.1% developing pancreatitis.^65^

**Table 9:** Extrarenal Manifestations Associated With aHUS

**Extrarenal manifestation**	**Reference**
Digital gangrene	220, 221, 222
Cerebral artery thrombosis/stenosis	222, 223, 224
Extracerebral artery stenosis	^223^
Cardiac involvement/myocardial infarction	51, 225
Ocular involvement	^226^
Pulmonary involvement	51, 94
Pancreatic involvement	^51^
Neurologic involvement	51, 94, 103

## Treatment

### Plasma Exchange

Until the beginning of this decade, plasma exchange (PE) was considered the gold standard for management of aHUS. The replacement of nonfunctioning complement proteins and removal of CFH autoantibodies and hyperfunctional complement components (eg, gain-of-function mutations) made PE a logical choice (reviewed in European^104^ and UK^105^ guidelines on aHUS treatment). The consensus-based guidelines recommended that PE should be commenced as soon as possible after diagnosis of aHUS, using 1 to 2 plasma volumes per session in adults and 50 to 100 mL/kg in children. Initially, PE should be performed daily and the dose titrated to control hemolysis. Once hemolysis has been controlled, PE can be withdrawn slowly, although individuals with genetic defects in the complement system are frequently plasma dependent and require long-term plasma therapy (weekly/biweekly) to maintain remission. Only once ADAMTS13 deficiency is excluded should eculizumab be considered. The price of eculizumab will mean that PE will remain the only currently available option in many countries.

### Eculizumab

Eculizumab, a recombinant humanized monoclonal antibody directed against C5, blocks the cleavage of C5 into its effector components C5a and C5b.^106^ Since the initial use of this complement inhibitor in aHUS in 2009,^107^ the effectiveness of eculizumab has been communicated in many case reports^107^, ^108^, ^109^, ^110^, ^111^, ^112^, ^113^, ^114^, ^115^, ^116^, ^117^, ^118^, ^119^, meeting abstracts^120^, ^121^, ^122^, ^123^, ^124^, ^125^, and a recently published prospective trial.^125a^

Eculizumab appears highly effective, with approximately 85% of patients becoming disease-free in both plasma-resistant and plasma-dependent aHUS (reviewed by Wong et al^126^). It is effective in patients with and without identified complement mutations. Similar to PE, the earlier eculizumab is commenced, the greater the preservation of kidney function. It has been suggested that eculizumab achieves better control of disease as witnessed by improvement in renal function after switching from PE and in rescuing plasma-resistant individuals.^126^ It should be noted, however, that a randomized trial of eculizumab against PE was not, and is unlikely to be, performed.

Treatment with eculizumab should begin as soon as Stx-HUS and ADAMTS13 deficiency can be eliminated. Current protocols suggest life-long treatment with eculizumab will be required, however, as we gain more clinical experience it is likely that there will be certain subgroups in whom the treatment can be stopped (eg, those with isolated MCP mutations). Eculizumab has been used safely during pregnancy in patients with paroxysmal nocturnal hemoglobinuria.^126^

Because host defense against encapsulated organisms is dependent on the ability to form a membrane attack complex (C5b-9), vaccination against *Neisseria meningitides* is required before treatment with eculizumab. However, because the current vaccine (tetravalent) does not cover the most prevalent strain (serogroup B), long-term prophylactic antibiotic cover also has been suggested.^126^

## Renal Transplantation in aHUS

The outcome of renal transplantation in patients with aHUS is poor. In one adult series of 71 renal transplants, the 5-year death-censored graft survival was only 51%, with a 7% mortality rate at 5 years.^99^ Graft failure is predominantly due to aHUS recurrence which occurs in 60% to 70% of patients,^99^, ^127^ and occurs early after transplant (70% in the first year after transplant^99^).

The outcome of renal transplantation is predicted largely by the underlying genetic abnormality. In individuals with mutations in *CFH* the recurrence rate is greater than 80%. Similarly, activating mutations in *C3* and *CFB* also have a high risk of renal recurrence. Initial studies all suggested that mutations in *CFI* carried a poor prognosis, although more recently one study failed to replicate these data.^99^ It is likely that this variation in data reflects the functional consequences of genetic variants in the different populations.

Unlike the complement proteins described earlier, *MCP* is membrane-tethered and is not a plasma protein. As such, a renal allograft will correct the complement defect and protect against aHUS. In keeping with this, the outcome after transplantation in those with mutations in *MCP* is much better, with a recurrence rate of only approximately 20%.^128^ It has been suggested that in those who do recur, an additional genetic predisposition such as an at-risk haplotype may be present^47^ or endothelial microchimerism may occur.^129^ In keeping with this, outcomes were worse in individuals with combined *MCP* mutations compared with patients with an isolated *MCP* change.^46^ Only one individual with a loss-of-function mutation in *THBD* has undergone transplantation to date and they had recurrent disease.^60^

It is perhaps not surprising that individuals with underlying genetic defects have a high recurrence rate because the post-transplant milieu provides the necessary disease triggers (eg, viral diseases, ischemia reperfusion injury, donor-specific antibodies, immunosuppressive drugs, and so forth) to cause endothelial cell damage and activation of the complement cascade. Calcineurin inhibitors (tacrolimus and cyclosporin), although consistently linked as a trigger for aHUS, were not shown to be associated significantly in two recent studies of aHUS recurrence post-transplant.^99^, ^127^ Mammalian target of rapamycin inhibitors (eg, sirolimus), however, have been reported to increase the risk of recurrence.^99^

Although plasma therapy has a low success rate in rescuing recurrent aHUS after renal transplantation, pre-emptive plasma exchange has been associated with a trend to decrease recurrence.^99^ Such a regimen may now be superseded by pre-emptive eculizumab.

## Autoantibodies

There is limited information regarding the outcome after transplantation in individuals with CFH autoantibodies. Two patients have been reported to have CFH autoantibodies and recurrent aHUS.^130^, ^131^ Two individuals had successful renal transplants using pre-emptive removal of *CFH* autoantibodies using rituximab and PE.^131^, ^132^ Five individuals with factor H autoantibodies have successfully undergone renal transplantation in the absence of specific therapy aimed at reducing *CFH* autoantibody titers with follow-up evaluation ranging from 2 to 17 years with no recurrence.^64^, ^67^ An additional confounding factor is that CFH autoantibodies frequently are found in association with other mutations. A pragmatic approach would be to use a regimen designed to remove CFH autoantibodies in those with a high titer.

## Living Related Transplantation

Live related transplantation is a particularly unattractive option in aHUS given the high recurrence rate and graft loss in the recipient. In addition, de novo aHUS has been recorded in four donors within a year of donation.^31^, ^133^, ^134^, ^135^ In at least one of these cases, a *CFH* mutation in the donor subsequently was reported.^31^ Although genotyping may reveal a known mutation in a family member, the additional presence of risk haplotypes and the fact that further genetic risk factors remain to be discovered makes it impossible to rule out subsequent aHUS for a donor.

## Liver/Kidney Transplantation

Because CFH, CFI C3, and CFB are synthesized predominantly in the liver; combined liver/kidney transplantation has been viewed as a mechanism to correct the underlying genetic deficiency of complement regulation. Twenty liver/kidney transplants have been reported in the literature to date.^100^, ^127^, ^136^, ^137^, ^138^, ^139^, ^140^, ^141^, ^142^, ^143^, ^144^, ^145^ The initial attempts were not successful because they did not include preoperative PE to correct the underlying complement defects.^136^, ^137^ In this setting, ischemia-reperfusion injury triggered the complement cascade, resulting in primary liver nonfunction.

Subsequently, Saland et al^144^ introduced perioperative PE in addition to aspirin and heparin, which has resulted in improved outcomes. Treatment guidelines for the use of liver-kidney transplantation in aHUS were recently proposed by a consensus conference.^146^ Despite this, of the 14 patients who underwent the revised procedure, 2 died (14% surgical mortality), and the availability of eculizumab may change the risk-benefit profile of this type of surgery.

## De Novo aHUS After Renal Transplant

The role of genetic defects in the complement system has also been shown in de novo aHUS after renal transplant. In a series of transplant patients whose initial diagnosis was not aHUS, 29% were seen to have a mutation in *CFI* or *CFH*.^147^ This may be an under-representation of the genetic predisposition because the genes for *CFB* and *C3* were not screened. The majority of these patients had chronic glomerulonephritis or malignant hypertension as their initial diagnosis. Mesangiocapillary glomerulonephritis/C3 glomerulopathy share many of the same complement risk factors as aHUS and the transformation from mesangiocapillary glomerulonephritis to aHUS is well recognized. Likewise, a patient presenting at ESRD with a biopsy picture of malignant hypertension is indistinguishable from aHUS. We would recommend that such patients also should be screened for complement mutations before transplantation.

More recently, a liver transplant recipient was shown to have developed de novo aHUS. Genetic analysis showed that the recipient carried a risk *MCP* haplotype but did not have a mutation. DNA from the donor liver, however, was shown to carry a mutant *CFH*. This is further evidence of the role of susceptibility genes in predisposing to post-transplant aHUS.^148^

## Eculizumab Use in Renal Transplantation

Eculizumab has also been used successfully in renal transplantation in those experiencing recurrent aHUS in a transplant^149^, ^150^, ^151^, ^152^, ^153^, ^154^ and as prophylaxis before transplantation in those with a known mutation.^155^, ^156^, ^157^, ^158^ In a recent review, Zuber et al^159^ reported that of 9 patients with complement mutations, 8 had successful renal transplants under eculizumab prophylaxis and 13 patients had successful salvage treatment for recurrent aHUS after transplant. In those individuals with ESRD secondary to complement-mediated aHUS requiring renal transplantation, it is probable that prophylactic eculizumab will become the treatment of choice.

## Summary

Overactivity of the AP is central to the pathogenesis of aHUS. Many underlying genetic complement predispositions have been described but disease only manifests after an environmental trigger. The understanding of the role of complement in the pathogenesis of aHUS has facilitated the introduction of a successful treatment in the form of the complement C5 inhibitor, eculizumab.

## References

[1] Kavanagh D., Richards A., Atkinson J. (2008). Complement regulatory genes and hemolytic uremic syndromes. Annu Rev Med.

[2] Besbas N., Karpman D., Landau D., Loirat C., Proesmans W., Remuzzi G. (2006). A classification of hemolytic uremic syndrome and thrombotic thrombocytopenic purpura and related disorders. Kidney Int.

[3] Constantinescu A.R., Bitzan M., Weiss L.S., Christen E., Kaplan B.S., Cnaan A. (2004). Non-enteropathic hemolytic uremic syndrome: causes and short-term course. Am J Kidney Dis.

[4] Fogo A., Kashgarian M. (2005).

[5] Ricklin D., Hajishengallis G., Yang K., Lambris J.D. (2010). Complement: a key system for immune surveillance and homeostasis. Nat Immunol.

[6] Kemper C., Atkinson J.P. (2007). T-cell regulation: with complements from innate immunity. Nat Rev Immunol.

[7] Warwicker P., Goodship T.H., Donne R.L., Pirson Y., Nicholls A., Ward R.M. (1998). Genetic studies into inherited and sporadic hemolytic uremic syndrome. Kidney Int.

[8] Richards A., Buddles M.R., Donne R.L., Kaplan B.S., Kirk E., Venning M.C. (2001). Factor H mutations in hemolytic uremic syndrome cluster in exons 18-20, a domain important for host cell recognition. Am J Hum Genet.

[9] Fan X., Yoshida Y., Honda S., Matsumoto M., Sawada Y., Hattori M. (2013). Analysis of genetic and predisposing factors in Japanese patients with atypical hemolytic uremic syndrome. Mol Immunol.

[10] Geerdink L.M., Westra D., van Wijk J.A., Dorresteijn E.M., Lilien M.R., Davin J.C. (2012). Atypical hemolytic uremic syndrome in children: complement mutations and clinical characteristics. Pediatr Nephrol.

[11] Maga T.K., Nishimura C.J., Weaver A.E., Frees K.L., Smith R.J. (2010). Mutations in alternative pathway complement proteins in American patients with atypical hemolytic uremic syndrome. Hum Mutat.

[12] Neumann H.P., Salzmann M., Bohnert-Iwan B., Mannuelian T., Skerka C., Lenk D. (2003). Haemolytic uraemic syndrome and mutations of the factor H gene: a registry-based study of German speaking countries. J Med Genet.

[13] Caprioli J., Bettinaglio P., Zipfel P.F., Amadei B., Daina E., Gamba S. (2001). The molecular basis of familial hemolytic uremic syndrome: mutation analysis of factor H gene reveals a hot spot in short consensus repeat 20. J Am Soc Nephrol.

[14] Perez-Caballero D., Gonzalez-Rubio C., Gallardo M.E., Vera M., Lopez-Trascasa M., Rodriguez de Cordoba S. (2001). Clustering of missense mutations in the C-terminal region of factor H in atypical hemolytic uremic syndrome. Am J Hum Genet.

[15] Dragon-Durey M.A., Fremeaux-Bacchi V., Loirat C., Blouin J., Niaudet P., Deschenes G. (2004). Heterozygous and homozygous factor h deficiencies associated with hemolytic uremic syndrome or membranoproliferative glomerulonephritis: report and genetic analysis of 16 cases. J Am Soc Nephrol.

[16] Richards A., Kavanagh D., Atkinson J.P. (2007). Inherited complement regulatory protein deficiency predisposes to human disease in acute injury and chronic inflammatory statesthe examples of vascular damage in atypical hemolytic uremic syndrome and debris accumulation in age-related macular degeneration. Adv Immunol.

[17] Schmidt C.Q., Herbert A.P., Kavanagh D., Gandy C., Fenton C.J., Blaum B.S. (2008). A new map of glycosaminoglycan and C3b binding sites on factor H. J Immunol.

[18] Ferreira V.P., Pangburn M.K., Cortes C. (2010). Complement control protein factor H: the good, the bad, and the inadequate. Mol Immunol.

[19] Clark S.J., Ridge L.A., Herbert A.P., Hakobyan S., Mulloy B., Wurzner R. (2013). Tissue-specific host recognition by complement factor H is mediated by differential activities of its glycoaminoglycans-binding regions. J Immunol.

[20] Weismann D., Hartvigsen K., Lauer N., Bennett K.L., Scholl H.P., Charbel Issa P. (2011). Complement factor H binds malondialdehyde epitopes and protects from oxidative stress. Nature.

[21] Sjoberg A.P., Trouw L.A., Clark S.J., Sjolander J., Heinegard D., Sim R.B. (2007). The factor H variant associated with age-related macular degeneration (His-384) and the non-disease-associated form bind differentially to C-reactive protein, fibromodulin, DNA, and necrotic cells. J Biol Chem.

[22] Laine M., Jarva H., Seitsonen S., Haapasalo K., Lehtinen M.J., Lindeman N. (2007). Y402H polymorphism of complement factor H affects binding affinity to C-reactive protein. J Immunol.

[23] Hakobyan S., Harris C.L., van den Berg C.W., Fernandez-Alonso M.C., de Jorge E.G., de Cordoba S.R. (2008). Complement factor H binds to denatured rather than to native pentameric C-reactive protein. J Biol Chem.

[24] Kopp A., Strobel S., Tortajada A., Rodriguez de Cordoba S., Sanchez-Corral P., Prohaszka Z (2012). Atypical hemolytic uremic syndrome-associated variants and autoantibodies impair binding of factor h and factor h-related protein 1 to pentraxin 3. J Immunol.

[25] Ferreira V.P., Herbert A.P., Cortes C., McKee K.A., Blaum B.S., Esswein S.T. (2009). The binding of factor H to a complex of physiological polyanions and C3b on cells is impaired in atypical hemolytic uremic syndrome. J Immunol.

[26] Abarrategui-Garrido C., Melgosa M., Pena-Carrion A., de Jorge E.G., de Cordoba S.R., Lopez-Trascasa M. (2008). Mutations in proteins of the alternative pathway of complement and the pathogenesis of atypical hemolytic uremic syndrome. Am J Kidney Dis.

[27] Vaziri-Sani F., Holmberg L., Sjoholm A.G., Kristoffersson A.C., Manea M., Fremeaux-Bacchi V. (2006). Phenotypic expression of factor H mutations in patients with atypical hemolytic uremic syndrome. Kidney Int.

[28] Stahl A.L., Vaziri-Sani F., Heinen S., Kristoffersson A.C., Gydell K.H., Raafat R. (2008). Factor H dysfunction in patients with atypical hemolytic uremic syndrome contributes to complement deposition on platelets and their activation. Blood.

[29] Pechtl I.C., Kavanagh D., McIntosh N., Harris C.L., Barlow P.N. (2011). Disease-associated N-terminal complement factor H mutations perturb cofactor and decay-accelerating activities. J Biol Chem.

[30] Tortajada A., Pinto S., Martinez-Ara J., Lopez-Trascasa M., Sanchez-Corral P., de Cordoba S.R. (2012). Complement factor H variants I890 and L1007 while commonly associated with atypical hemolytic uremic syndrome are polymorphisms with no functional significance. Kidney Int.

[31] Heinen S., Sanchez-Corral P., Jackson M.S., Strain L., Goodship J.A., Kemp E.J. (2006). De novo gene conversion in the RCA gene cluster (1q32) causes mutations in complement factor H associated with atypical hemolytic uremic syndrome. Hum Mutat.

[32] Venables J.P., Strain L., Routledge D., Bourn D., Powell H.M., Warwicker P. (2006). Atypical haemolytic uraemic syndrome associated with a hybrid complement gene. PLoS Med.

[33] Francis N.J., McNicholas B., Awan A., Waldron M., Reddan D., Sadlier D. (2012). A novel hybrid CFH/CFHR3 gene generated by a microhomology-mediated deletion in familial atypical hemolytic uremic syndrome. Blood.

[34] Pickering M.C., de Jorge E.G., Martinez-Barricarte R., Recalde S., Garcia-Layana A., Rose K.L. (2007). Spontaneous hemolytic uremic syndrome triggered by complement factor H lacking surface recognition domains. J Exp Med.

[35] Richards A., Kavanagh D. (2009). Pathogenesis of thrombotic microangiopathy: insights from animal models. Nephron Exp Nephrol.

[36] Goicoechea de Jorge E., Macor P., Paixao-Cavalcante D., Rose K.L., Tedesco F., Cook H.T. (2011). The development of atypical hemolytic uremic syndrome depends on complement C5. J Am Soc Nephrol.

[37] Fremeaux-Bacchi V., Dragon-Durey M.A., Blouin J., Vigneau C., Kuypers D., Boudailliez B. (2004). Complement factor I: a susceptibility gene for atypical haemolytic uraemic syndrome. J Med Genet.

[38] Kavanagh D., Kemp E.J., Mayland E., Winney R.J., Duffield J.S., Warwick G. (2005). Mutations in complement factor I predispose to development of atypical hemolytic uremic syndrome. J Am Soc Nephrol.

[39] Kavanagh D., Richards A., Noris M., Hauhart R., Liszewski M.K., Karpman D. (2008). Characterization of mutations in complement factor I (CFI) associated with hemolytic uremic syndrome. Mol Immunol.

[40] Caprioli J., Noris M., Brioschi S., Pianetti G., Castelletti F., Bettinaglio P. (2006). Genetics of HUS: the impact of MCP, CFH, and IF mutations on clinical presentation, response to treatment, and outcome. Blood.

[41] Nilsson S.C., Kalchishkova N., Trouw L.A., Fremeaux-Bacchi V., Villoutreix B.O., Blom A.M. (2010). Mutations in complement factor I as found in atypical hemolytic uremic syndrome lead to either altered secretion or altered function of factor I. Eur J Immunol.

[42] Nilsson S.C., Karpman D., Vaziri-Sani F., Kristoffersson A.C., Salomon R., Provot F. (2007). A mutation in factor I that is associated with atypical hemolytic uremic syndrome does not affect the function of factor I in complement regulation. Mol Immunol.

[43] Westra D., Volokhina E., van der Heijden E., Vos A., Huigen M., Jansen J. (2010). Genetic disorders in complement (regulating) genes in patients with atypical haemolytic uraemic syndrome (aHUS). Nephrol Dial Transplant.

[44] Sullivan M., Erlic Z., Hoffmann M.M., Arbeiter K., Patzer L., Budde K. (2010). Epidemiological approach to identifying genetic predispositions for atypical hemolytic uremic syndrome. Ann Hum Genet.

[45] Richards A., Kemp E.J., Liszewski M.K., Goodship J.A., Lampe A.K., Decorte R. (2003). Mutations in human complement regulator, membrane cofactor protein (CD46), predispose to development of familial hemolytic uremic syndrome. Proc Natl Acad Sci U S A.

[46] Bresin E., Rurali E., Caprioli J., Sanchez-Corral P., Fremeaux-Bacchi V., Rodriguez de Cordoba S. (2013). Combined complement gene mutations in atypical hemolytic uremic syndrome influence clinical phenotype. J Am Soc Nephrol.

[47] Fremeaux-Bacchi V., Moulton E.A., Kavanagh D., Dragon-Durey M.A., Blouin J., Caudy A. (2006). Genetic and functional analyses of membrane cofactor protein (CD46) mutations in atypical hemolytic uremic syndrome. J Am Soc Nephrol.

[48] Richards A., Liszewski K.M., Kavanagh D., Fang C.J., Moulton E., Fremeaux-Bacchi V. (2007). Implications of the initial mutations in membrane cofactor protein (MCP; CD46) leading to atypical hemolytic uremic syndrome. Mol Immunol.

[49] Fremeaux-Bacchi V., Fakhouri F., Garnier A., Bienaime F., Dragon-Durey M.A., Ngo S. (2013). Genetics and outcome of atypical hemolytic uremic syndrome: a nationwide french series comparing children and adults. Clin J Am Soc Nephrol.

[50] Fremeaux-Bacchi V., Miller E.C., Liszewski M.K., Strain L., Blouin J., Brown A.L. (2008). Mutations in complement C3 predispose to development of atypical hemolytic uremic syndrome. Blood.

[51] Noris M., Caprioli J., Bresin E., Mossali C., Pianetti G., Gamba S. (2010). Relative role of genetic complement abnormalities in sporadic and familial aHUS and their impact on clinical phenotype. Clin J Am Soc Nephrol.

[52] Sartz L., Olin A.I., Kristoffersson A.C., Stahl A.L., Johansson M.E., Westman K. (2012). A novel C3 mutation causing increased formation of the C3 convertase in familial atypical hemolytic uremic syndrome. J Immunol.

[53] Lhotta K., Janecke A.R., Scheiring J., Petzlberger B., Giner T., Fally V. (2009). A large family with a gain-of-function mutation of complement C3 predisposing to atypical hemolytic uremic syndrome, microhematuria, hypertension and chronic renal failure. Clin J Am Soc Nephrol.

[54] Roumenina L.T., Frimat M., Miller E.C., Provot F., Dragon-Durey M.A., Bordereau P. (2012). A prevalent C3 mutation in aHUS patients causes a direct C3 convertase gain of function. Blood.

[55] Roumenina L.T., Jablonski M., Hue C., Blouin J., Dimitrov J.D., Dragon-Durey M.A. (2009). Hyperfunctional C3 convertase leads to complement deposition on endothelial cells and contributes to atypical hemolytic uremic syndrome. Blood.

[56] Goicoechea de Jorge E., Harris C.L., Esparza-Gordillo J., Carreras L., Arranz E.A., Garrido C.A. (2007). Gain-of-function mutations in complement factor B are associated with atypical hemolytic uremic syndrome. Proc Natl Acad Sci U S A.

[57] Kavanagh D., Kemp E.J., Richards A., Burgess R.M., Mayland E., Goodship J.A. (2006). Does complement factor B have a role in the pathogenesis of atypical HUS?. Mol Immunol.

[58] Tawadrous H., Maga T., Sharma J., Kupferman J., Smith R.J., Schoeneman M. (2010). A novel mutation in the complement factor B gene (CFB) and atypical hemolytic uremic syndrome. Pediatr Nephrol.

[59] Weiler H., Isermann B.H. (2003). Thrombomodulin. J Thromb Haemost.

[60] Delvaeye M., Noris M., De Vriese A., Esmon C.T., Esmon N.L., Ferrell G. (2009). Thrombomodulin mutations in atypical hemolytic-uremic syndrome. N Engl J Med.

[61] Westra D., Vernon K.A., Volokhina E.B., Pickering M.C., van de Kar N.C., van den Heuvel L.P. (2012). Atypical hemolytic uremic syndrome and genetic aberrations in the complement factor H-related 5 gene. J Hum Genet.

[62] Monteferrante G., Brioschi S., Caprioli J., Pianetti G., Bettinaglio P., Bresin E. (2007). Genetic analysis of the complement factor H related 5 gene in haemolytic uraemic syndrome. Mol Immunol.

[63] Stahl A.L., Kristoffersson A., Olin A.I., Olsson M.L., Roodhooft A.M., Proesmans W. (2009). A novel mutation in the complement regulator clusterin in recurrent hemolytic uremic syndrome. Mol Immunol.

[64] Abarrategui-Garrido C., Martinez-Barricarte R., Lopez-Trascasa M., de Cordoba S.R., Sanchez-Corral P. (2009). Characterization of complement factor H-related (CFHR) proteins in plasma reveals novel genetic variations of CFHR1 associated with atypical hemolytic uremic syndrome. Blood.

[65] Dragon-Durey M.A., Sethi S.K., Bagga A., Blanc C., Blouin J., Ranchin B. (2010). Clinical features of anti-factor H autoantibody-associated hemolytic uremic syndrome. J Am Soc Nephrol.

[66] Jozsi M., Licht C., Strobel S., Zipfel S.L., Richter H., Heinen S. (2008). Factor H autoantibodies in atypical hemolytic uremic syndrome correlate with CFHR1/CFHR3 deficiency. Blood.

[67] Moore I., Strain L., Pappworth I., Kavanagh D., Barlow P.N., Herbert A.P. (2010). Association of factor H autoantibodies with deletions of CFHR1, CFHR3, CFHR4, and with mutations in CFH, CFI, CD46, and C3 in patients with atypical hemolytic uremic syndrome. Blood.

[68] Foltyn Zadura A., Zipfel P.F., Bokarewa M.I., Sturfelt G., Jonsen A., Nilsson S.C. (2012). Factor H autoantibodies and deletion of complement factor H-related protein-1 in rheumatic diseases in comparison to atypical hemolytic uremic syndrome. Arthritis Res Ther.

[69] Hofer J., Janecke A.R., Zimmerhackl L.B., Riedl M., Rosales A., Giner T. (2013). Complement factor H-related protein 1 deficiency and factor H antibodies in pediatric patients with atypical hemolytic uremic syndrome. Clin J Am Soc Nephrol.

[70] Zipfel P.F., Edey M., Heinen S., Jozsi M., Richter H., Misselwitz J. (2007). Deletion of complement factor H-related genes CFHR1 and CFHR3 is associated with atypical hemolytic uremic syndrome. PLoS Genet.

[71] Abarrategui-Garrido C., Martinez-Barricarte R., Lopez-Trascasa M., Rodriguez de Cordoba S., Sanchez-Corral P. (2009). Characterization of complement factor H-related (CFHR) proteins in plasma reveals novel genetic variations of CFHR1 associated with atypical hemolytic uremic syndrome. Blood.

[72] Moore I., Strain L., Pappworth I., Kavanagh D., Barlow P.N., Herbert A.P. (2010). Association of factor H autoantibodies with deletions of CFHR1, CFHR3, CFHR4 and with mutations in CFH, CFI, CD46 and C3 in patients with atypical haemolytic uraemic syndrome. Blood.

[73] Dragon-Durey M.A., Blanc C., Marliot F., Loirat C., Blouin J., Sautes-Fridman C. (2009). The high frequency of complement factor H related CFHR1 gene deletion is restricted to specific subgroups of patients with atypical haemolytic uraemic syndrome. J Med Genet.

[74] Strobel S., Abarrategui-Garrido C., Fariza-Requejo E., Seeberger H., Sanchez-Corral P., Jozsi M. (2011). Factor H-related protein 1 neutralizes anti-factor H autoantibodies in autoimmune hemolytic uremic syndrome. Kidney Int.

[75] Jozsi M., Strobel S., Dahse H.M., Liu W.S., Hoyer P.F., Oppermann M. (2007). Anti factor H autoantibodies block C-terminal recognition function of factor H in hemolytic uremic syndrome. Blood.

[76] Dragon-Durey M.A., Loirat C., Cloarec S., Macher M.A., Blouin J., Nivet H. (2005). Anti-factor H autoantibodies associated with atypical hemolytic uremic syndrome. J Am Soc Nephrol.

[77] Blanc C., Roumenina L.T., Ashraf Y., Hyvarinen S., Sethi S.K., Ranchin B. (2012). Overall neutralization of complement factor H by autoantibodies in the acute phase of the autoimmune form of atypical hemolytic uremic syndrome. J Immunol.

[78] Kavanagh D., Pappworth I.Y., Anderson H., Hayes C.M., Moore I., Hunze E.M. (2012). Factor I autoantibodies in patients with atypical hemolytic uremic syndrome: disease-associated or an epiphenomenon?. Clin J Am Soc Nephrol.

[79] Caprioli J., Castelletti F., Bucchioni S., Bettinaglio P., Bresin E., Pianetti G. (2003). Complement factor H mutations and gene polymorphisms in haemolytic uraemic syndrome: the C-257T, the A2089G and the G2881T polymorphisms are strongly associated with the disease. Hum Mol Genet.

[80] Esparza-Gordillo J., Goicoechea de Jorge E., Buil A., Carreras Berges L., Lopez-Trascasa M., Sanchez-Corral P. (2005). Predisposition to atypical hemolytic uremic syndrome involves the concurrence of different susceptibility alleles in the regulators of complement activation gene cluster in 1q32. Hum Mol Genet.

[81] Fremeaux-Bacchi V., Kemp E.J., Goodship J.A., Dragon-Durey M.A., Strain L., Loirat C. (2005). The development of atypical haemolytic-uraemic syndrome is influenced by susceptibility factors in factor H and membrane cofactor protein: evidence from two independent cohorts. J Med Genet.

[82] Ermini L., Goodship T.H., Strain L., Weale M.E., Sacks S.H., Cordell H.J. (2012). Common genetic variants in complement genes other than CFH, CD46 and the CFHRs are not associated with aHUS. Mol Immunol.

[83] Harris C.L., Heurich M., Rodriguez de Cordoba S., Morgan B.P. (2012). The complotype: dictating risk for inflammation and infection. Trends Immunol.

[84] Tortajada A., Montes T., Martinez-Barricarte R., Morgan B.P., Harris C.L. (2009). de Cordoba SR. The disease-protective complement factor H allotypic variant Ile62 shows increased binding affinity for C3b and enhanced cofactor activity. Hum Mol Genet.

[85] Hocking H.G., Herbert A.P., Kavanagh D., Soares D.C., Ferreira V.P., Pangburn M.K. (2008). Structure of the N-terminal region of complement factor H and conformational implications of disease-linked sequence variations. J Biol Chem.

[86] Esparza-Gordillo J., Jorge E.G., Garrido C.A., Carreras L., Lopez-Trascasa M., Sanchez-Corral P. (2006). Insights into hemolytic uremic syndrome: segregation of three independent predisposition factors in a large, multiple affected pedigree. Mol Immunol.

[87] Frimat M., Roumenina L., Tabarin F., Halbwachs-Mecarelli L., Fremeaux-Bacchi V. (2012). Membrane cofactor protein (MCP) haplotype, which predisposes to atypical hemolytic and uremic syndrome, has no consequence on neutrophils and endothelial cells MCP levels or on HUVECs ability to activate complement. Immunobiology.

[88] Blom A.M., Bergstrom F., Edey M., Diaz-Torres M., Kavanagh D., Lampe A. (2008). A novel non-synonymous polymorphism (p.Arg240His) in C4b-binding protein is associated with atypical hemolytic uremic syndrome and leads to impaired alternative pathway cofactor activity. J Immunol.

[89] Martinez-Barricarte R., Goicoechea de Jorge E., Montes T., Layana A.G., Rodriguez de Cordoba S. (2009). Lack of association between polymorphisms in C4b-binding protein and atypical haemolytic uraemic syndrome in the Spanish population. Clin Exp Immunol.

[90] Kavanagh D., Goodship T. (2010). Genetics and complement in atypical HUS. Pediatr Nephrol.

[91] Sullivan M., Rybicki L.A., Winter A., Hoffmann M.M., Reiermann S., Linke H. (2011). Age-related penetrance of hereditary atypical hemolytic uremic syndrome. Ann Hum Genet.

[92] Cruzado J.M., de Cordoba S.R., Melilli E., Bestard O., Rama I., Sanchez-Corral P. (2009). Successful renal transplantation in a patient with atypical hemolytic uremic syndrome carrying mutations in both factor I and MCP. Am J Transplant.

[93] Bienaime F., Dragon-Durey M.A., Regnier C.H., Nilsson S.C., Kwan W.H., Blouin J. (2010). Mutations in components of complement influence the outcome of factor I-associated atypical hemolytic uremic syndrome. Kidney Int.

[94] Sellier-Leclerc A.L., Fremeaux-Bacchi V., Dragon-Durey M.A., Macher M.A., Niaudet P., Guest G. (2007). Differential impact of complement mutations on clinical characteristics in atypical hemolytic uremic syndrome. J Am Soc Nephrol.

[95] Edey M.M., Mead P.A., Saunders R.E., Strain L., Perkins S.J., Goodship T.H. (2008). Association of a factor H mutation with hemolytic uremic syndrome following a diarrheal illness. Am J Kidney Dis.

[96] Fakhouri F., Roumenina L., Provot F., Sallee M., Caillard S., Couzi L. (2010). Pregnancy-associated hemolytic uremic syndrome revisited in the era of complement gene mutations. J Am Soc Nephrol.

[97] Goodship T.H., Kavanagh D. (2010). Pulling the trigger in atypical hemolytic uremic syndrome: the role of pregnancy. J Am Soc Nephrol.

[98] Fakhouri F., Roumenina L., Provot F., Sallee M., Caillard S., Couzi L. (2010). Pregnancy-associated hemolytic uremic syndrome revisited in the era of complement gene mutations. J Am Soc Nephrol.

[99] Le Quintrec M., Zuber J., Moulin B., Kamar N., Jablonski M., Lionet A. (2013). Complement genes strongly predict recurrence and graft outcome in adult renal transplant recipients with atypical hemolytic and uremic syndrome. Am J Transplant.

[100] Loirat C., Fremeaux-Bacchi V. (2011). Atypical hemolytic uremic syndrome. Orphanet J Rare Dis.

[101] Kavanagh D., Richards A., Fremeaux-Bacchi V., Noris M., Goodship T., Remuzzi G. (2007). Screening for complement system abnormalities in patients with atypical hemolytic uremic syndrome. Clin J Am Soc Nephrol.

[102] Kavanagh D., Anderson H.E. (2012). Interpretation of genetic variants of uncertain significance in atypical hemolytic uremic syndrome. Kidney Int.

[103] Koehl B., Boyer O., Biebuyck-Gouge N., Kossorotoff M., Fremeaux-Bacchi V., Boddaert N. (2010). Neurological involvement in a child with atypical hemolytic uremic syndrome. Pediatr Nephrol.

[104] Ariceta G., Besbas N., Johnson S., Karpman D., Landau D., Licht C. (2009). Guideline for the investigation and initial therapy of diarrhea-negative hemolytic uremic syndrome. Pediatr Nephrol.

[105] Taylor C.M., Machin S., Wigmore S.J., Goodship T.H. (2010). Clinical practice guidelines for the management of atypical haemolytic uraemic syndrome in the United Kingdom. Br J Haematol.

[106] Rother R.P., Rollins S.A., Mojcik C.F., Brodsky R.A., Bell L. (2007). Discovery and development of the complement inhibitor eculizumab for the treatment of paroxysmal nocturnal hemoglobinuria. Nat Biotechnol.

[107] Gruppo R.A., Rother R.P. (2009). Eculizumab for congenital atypical hemolytic-uremic syndrome. N Engl J Med.

[108] Ariceta G., Arrizabalaga B., Aguirre M., Morteruel E., Lopez-Trascasa M. (2012). Eculizumab in the treatment of atypical hemolytic uremic syndrome in infants. Am J Kidney Dis.

[109] Dorresteijn E.M., van de Kar NCAJ, Cransberg K. (2012). Eculizumab as rescue therapy for atypical hemolytic uremic syndrome with normal platelet count. Pediatr Nephrol.

[110] Lapeyraque A.-L., Fremeaux-Bacchi V., Robitaille P. (2011). Efficacy of eculizumab in a patient with factor-H-associated atypical hemolytic uremic syndrome. Pediatr Nephrol.

[111] Ohanian M., Cable C., Halka K. (2011). Eculizumab safely reverses neurologic impairment and eliminates need for dialysis in severe atypical hemolytic uremic syndrome. Clin Pharmacol.

[112] Prescott H.C., Wu H.M., Cataland S.R., Baiocchi R.A. (2010). Eculizumab therapy in an adult with plasma exchange-refractory atypical hemolytic uremic syndrome. Am J Hematol.

[113] Tschumi S., Gugger M., Bucher B.S., Riedl M., Simonetti G.D. (2011). Eculizumab in atypical hemolytic uremic syndrome: long-term clinical course and histological findings. Pediatr Nephrol.

[114] Koese O., Zimmerhackl L.-B., Jungraithmayr T., Mache C., Nuernberger J. (2010). New treatment options for atypical hemolytic uremic syndrome with the complement inhibitor eculizumab. Semin Thromb Hemost.

[115] Kim JJ, Waller SC, Reid CJ. Eculizumab in atypical haemolytic-uraemic syndrome allows cessation of plasma exchange and dialysis. Clin Kidney J. 2012;5:34-6 10.1093/ndtplus/sfr174 PMC4400463 26069744

[116] Malina M. Peripheral gangrene in children with atypical hemolytic uremic syndrome. Paper presented at: 44th Annual Meeting of the European Society for Pediatric Nephrology 14 Sept 2011 Dubrovnik, Croatia

[117] Fremont OT. Eculizumab treatment for aHUS in a child with positive family history. Paper presented at: 42nd Annual Meeting of the American Society of Nephrology. 27 Oct 2009 San Diego CA

[118] Garjau M. (2012). Early treatment with eculizumab may be beneficial in atypical haemolytic uraemic syndrome. Clin Kidney J.

[119] Haffner K. Effective eculizumab therapy of familiar atypical HUS in a 4 year old patient Paper presented at: 2nd International Conference on HUS, MPGN and PNH 13 June 2010 Innsbruck, Austria

[120] Muus P. Safety and efficacy of eculizumab in aHUS patients on chronic plasma therapy: Interim analysis of a phase II trial. Paper presented at: 43rd Annual Meeting of the American Society of Nephrology November 16, 2010; Denver, CO

[121] Licht C. Phase II study of eculizumab in patients with atypical HUS receiving chronic plasma exchange/infusion. Paper presented at 44th Annual Meeting of the American Society of Nephrology November 8, 2011; Philadelphia, PA

[122] Greenbaum LA. Eculizumab is an effective long-term treatment in patients with atypical haemolytic uremic syndrome previously receiving chronic plasma exchange/infusion: extension study results. Presented at the 53rd Annual Meeting of the American Society of Hematology; December 10, 2011; San Diego, CA

[123] Muus PM. Safety and efficacy of eculizumab in aHUS patients on chronic plasma therapy: interim analysis of a phase II trial. Presented at the 43rd Annual Meeting of the American Society of Nephrology; November 16, 2010; Denver, CO

[124] Greenbaum LA. Continued improvement in renal function with sustained eculizumab in patients with atypical HUS resistant to plasma exchange/infusion. Paper presented at 44th Annual Meeting of the American Society of Nephrology November 8, 2011; Philadelphia, PA

[125] Licht C. Eculizumab is an effective long-term treatment in patients with atypical haemolytic uremic syndrome previously receiving chronic plasma exchange/infusion: Extension study results. Presented at the 53rd Annual Meeting of the American Society of Hematology; December 10, 2011; San Diego, CA

[bib125a] Legendre C.M., Licht C., Muus P., Greenbaum L.A., Babu S., Bedrosian C. (2013). Terminal complement inhibitor eculizumab in atypical hemolytic-uremic syndrome. New Engl J Med.

[126] Wong EK, Goodship TH, Kavanagh D. Complement therapy in atypical haemolytic uraemic syndrome (aHUS). Mol Immunol. 2013;56:199-212 10.1016/j.molimm.2013.05.224 PMC3899040 23810412

[127] Bresin E., Daina E., Noris M., Castelletti F., Stefanov R., Hill P. (2006). Outcome of renal transplantation in patients with non-Shiga toxin-associated hemolytic uremic syndrome: prognostic significance of genetic background. Clin J Am Soc Nephrol.

[128] Loirat C., Fremeaux-Bacchi V. (2008). Hemolytic uremic syndrome recurrence after renal transplantation. Pediatr Transplant.

[129] Fremeaux-Bacchi V., Arzouk N., Ferlicot S., Charpentier B., Snanoudj R., Durrbach A. (2007). Recurrence of HUS due to CD46/MCP mutation after renal transplantation: a role for endothelial microchimerism. Am J Transplant.

[130] Lorcy N., Rioux-Leclercq N., Lombard M.L., Le Pogamp P., Vigneau C. (2011). Three kidneys, two diseases, one antibody?. Nephrol Dial Transplant.

[131] Le Quintrec M., Zuber J., Noel L.H., Thervet E., Fremeaux-Bacchi V., Niaudet P. (2009). Anti-factor H autoantibodies in a fifth renal transplant recipient with atypical hemolytic and uremic syndrome. Am J Transplant.

[132] Kwon T., Dragon-Durey M.A., Macher M.A., Baudouin V., Maisin A., Peuchmaur M. (2008). Successful pre-transplant management of a patient with anti-factor H autoantibodies-associated haemolytic uraemic syndrome. Nephrol Dial Transplant.

[133] Donne R.L., Abbs I., Barany P., Elinder C.G., Little M., Conlon P. (2002). Recurrence of hemolytic uremic syndrome after live related renal transplantation associated with subsequent de novo disease in the donor. Am J Kidney Dis.

[134] Bergstein J., Michael A., Kellstrand C., Simmons R., Najarian J. (1974). Hemolytic-uremic syndrome in adult sisters. Transplantation.

[135] Kaplan B.S., Papadimitriou M., Brezin J.H., Tomlanovich S.J., Zulkharnain (1997). Renal transplantation in adults with autosomal recessive inheritance of hemolytic uremic syndrome. Am J Kidney Dis.

[136] Remuzzi G., Ruggenenti P., Codazzi D., Noris M., Caprioli J., Locatelli G. (2002). Combined kidney and liver transplantation for familial haemolytic uraemic syndrome. Lancet.

[137] Remuzzi G., Ruggenenti P., Colledan M., Gridelli B., Bertani A., Bettinaglio P. (2005). Hemolytic uremic syndrome: a fatal outcome after kidney and liver transplantation performed to correct factor h gene mutation. Am J Transplant.

[138] Cheong H.I., Lee B.S., Kang H.G., Hahn H., Suh K.S., Ha I.S. (2004). Attempted treatment of factor H deficiency by liver transplantation. Pediatr Nephrol.

[139] Jalanko H., Peltonen S., Koskinen A., Puntila J., Isoniemi H., Holmberg C. (2008). Successful liver-kidney transplantation in two children with aHUS caused by a mutation in complement factor H. Am J Transplant.

[140] Saland J.M., Shneider B.L., Bromberg J.S., Shi P.A., Ward S.C., Magid M.S. (2009). Successful split liver-kidney transplant for factor H associated hemolytic uremic syndrome. Clin J Am Soc Nephrol.

[141] Haller W., Milford D.V., Goodship T.H., Sharif K., Mirza D.F., McKiernan P.J. (2010). Successful isolated liver transplantation in a child with atypical hemolytic uremic syndrome and a mutation in complement factor H. Am J Transplant.

[142] Wilson C., Torpey N., Jaques B., Strain L., Talbot D., Manas D. (2011). Successful simultaneous liver-kidney transplant in an adult with atypical hemolytic uremic syndrome associated with a mutation in complement factor H. Am J Kidney Dis.

[143] Sanchez-Corral P., Melgosa M. (2010). Advances in understanding the aetiology of atypical haemolytic uraemic syndrome. Br J Haematol.

[144] Saland J.M., Emre S.H., Shneider B.L., Benchimol C., Ames S., Bromberg J.S. (2006). Favorable long-term outcome after liver-kidney transplant for recurrent hemolytic uremic syndrome associated with a factor H mutation. Am J Transplant.

[145] Kavanagh D., Richards A., Goodship T., Jalanko H. (2010). Transplantation in atypical hemolytic uremic syndrome. Semin Thromb Hemost.

[146] Saland J.M., Ruggenenti P., Remuzzi G. (2009). Liver-kidney transplantation to cure atypical hemolytic uremic syndrome. J Am Soc Nephrol.

[147] Le Quintrec M., Lionet A., Kamar N., Karras A., Barbier S., Buchler M. (2008). Complement mutation-associated de novo thrombotic microangiopathy following kidney transplantation. Am J Transplant.

[148] Brown J.H., Tellez J., Wilson V., Mackie I.J., Scully M., Tredger M.M. (2012). Postpartum aHUS secondary to a genetic abnormality in factor H acquired through liver transplantation. Am J Transplant.

[149] Nuernberger J., Witzke O., Saez A.O., Vester U., Baba H.A., Kribben A. (2009). Eculizumab for atypical hemolytic-uremic syndrome. N Engl J Med.

[150] Al-Akash S.I., Almond P.S., Savell V.H., Gharaybeh S.I., Hogue C. (2011). Eculizumab induces long-term remission in recurrent post-transplant HUS associated with C3 gene mutation. Pediatr Nephrol.

[151] Chatelet V, Fremeaux-Bacchi V, Lobbedez T, Ficheux M, de Ligny BH. Safety and long-term efficacy of eculizumab in a renal transplant patient with recurrent atypical hemolytic-uremic syndrome. Am J Transplant. 2009;9:2644-5 10.1111/j.1600-6143.2009.02817.x 19775316

[152] Chatelet V., Lobbedez T., Fremeaux-Bacchi V., Ficheux M., Ryckelynck J.P., de Ligny BH. (2010). Eculizumab: safety and efficacy after 17 months of treatment in a renal transplant patient with recurrent atypical hemolytic-uremic syndrome: case report. Transplant Proc.

[153] Larrea CF-d Cofan F., Oppenheimer F., Campistol J.M., Escolar G., Lozano M. (2010). Efficacy of eculizumab in the treatment of recurrent atypical hemolytic-uremic syndrome after renal transplantation. Transplantation.

[154] Zuber J., Le Quintrec M., Sberro-Soussan R., Loirat C., Fremeaux-Bacchi V., Legendre C. (2011). New insights into postrenal transplant hemolytic uremic syndrome. Nat Rev Nephrol.

[155] Nester C., Stewart Z., Myers D., Jetton J, Nair R, Reed A (2011). Pre-emptive eculizumab and plasmapheresis for renal transplant in atypical hemolytic uremic syndrome. Clin J Am Soc Nephrol.

[156] Krid S., Roumenina L., Beury D., Charbit M., Boyer O., Fremeaux-Bacchi V. (2012). Renal transplantation under prophylactic eculizumab in atypical hemolytic uremic syndrome with CFH/CFHR1 hybrid protein. Am J Transplant.

[157] Weitz M., Amon O., Bassler D., Koenigsrainer A., Nadalin S. (2011). Prophylactic eculizumab prior to kidney transplantation for atypical hemolytic uremic syndrome. Pediatr Nephrol.

[158] Zimmerhackl L.B., Hofer J., Cortina G., Mark W., Wurzner R., Jungraithmayr T.C. (2010). Prophylactic eculizumab after renal transplantation in atypical hemolytic-uremic syndrome. N Engl J Med.

[159] Zuber J., Le Quintrec M., Krid S., Bertoye C., Gueutin V., Lahoche A., French Study Group (2012). Atypical HUS. Eculizumab for atypical hemolytic uremic syndrome recurrence in renal transplantation. Am J Transplant.

[160] Roversi P., Johnson S., Caesar J.J., McLean F., Leath K.J., Tsiftsoglou S.A. (2011). Structural basis for complement factor I control and its disease-associated sequence polymorphisms. Proc Natl Acad Sci U S A.

[161] Sugimoto T., Ogawa N., Aoyama M., Sakaguchi M., Isshiki K., Kanasaki M. (2007). Haemolytic uraemic syndrome complicated with norovirus-associated gastroenteritis. Nephrol Dial Transplant.

[162] Lee B.H., Kwak S.H., Shin J.I., Lee S.H., Choi H.J., Kang H.G. (2009). Atypical hemolytic uremic syndrome associated with complement factor H autoantibodies and CFHR1/CFHR3 deficiency. Pediatr Res.

[163] Carter J.E., Cimolai N. (1996). Hemolytic-uremic syndrome associated with acute Campylobacter upsaliensis gastroenteritis. Nephron.

[164] Alvarado A.S., Brodsky S.V., Nadasdy T., Singh N. (2013). Hemolytic uremic syndrome associated with Clostridium difficile infection. Clin Nephrol.

[165] Berner R., Krause M.F., Gordjani N., Zipfel P.F., Boehm N., Krueger M. (2002). Hemolytic uremic syndrome due to an altered factor H triggered by neonatal pertussis. Pediatr Nephrol.

[166] Waters A.M., Kerecuk L., Luk D., Haq M.R., Fitzpatrick M.M., Gilbert R.D. (2007). Hemolytic uremic syndrome associated with invasive pneumococcal disease: the United Kingdom experience. J Pediatr.

[167] Chand D.H., Brady R.C., Bissler J.J. (2001). Hemolytic uremic syndrome in an adolescent with Fusobacterium necrophorum bacteremia. Am J Kidney Dis.

[168] Kwon T., Belot A., Ranchin B., Baudouin V., Fremeaux-Bacchi V., Dragon-Durey M.A. (2009). Varicella as a trigger of atypical haemolytic uraemic syndrome associated with complement dysfunction: two cases. Nephrol Dial Transplant.

[169] Waiser J., Budde K., Rudolph B., Ortner M.A., Neumayer H.H. (1999). De novo hemolytic uremic syndrome postrenal transplant after cytomegalovirus infection. Am J Kidney Dis.

[170] Bento D., Mapril J., Rocha C., Marchbank K.J., Kavanagh D., Barge D. (2010). Triggering of atypical hemolytic uremic syndrome by influenza A (H1N1). Ren Fail.

[171] Tagle M., Barriga J.A., Gutierrez S., Valdez L.M., Castle J., Antunez De Mayolo A. (2004). [Relapsing viral hepatitis type A complicated with renal failure]. Rev Gastroenterol Peru.

[172] Baid S., Pascual M., Williams W.W., Tolkoff-Rubin N., Johnson S.M., Collins B. (1999). Renal thrombotic microangiopathy associated with anticardiolipin antibodies in hepatitis C-positive renal allograft recipients. J Am Soc Nephrol.

[173] Benitez M., Boto A., Colchero J., Fernandez-Giron F., Rodriguez P., Paralle M. (1997). Haemolytic-uraemic syndrome in a patient infected by HIV. Nephrol Dial Transplant.

[174] Austin T.W., Ray C.G., Coxsackie virus group (1973). B infections and the hemolytic-uremic syndrome. J Infect Dis.

[175] Watanabe T. (2004). Hemolytic uremic syndrome associated with Epstein-Barr virus infection. Pediatr Nephrol.

[176] Wiersinga W.J., Scheepstra C.G., Kasanardjo J.S., de Vries PJ, Zaaijer H, Geerlings SE. (2006). Dengue fever-induced hemolytic uremic syndrome. Clin Infect Dis.

[177] Matsuda Y., Hara J., Miyoshi H., Osugi Y., Fujisaki H., Takai K. (1999). Thrombotic microangiopathy associated with reactivation of human herpesvirus-6 following high-dose chemotherapy with autologous bone marrow transplantation in young children. Bone Marrow Transplant.

[178] Hartel C., Herz A., Vieth S., Lensing C., Schultz C. (2007). Renal complications associated with human parvovirus B19 infection in early childhood. Klin Padiatr.

[179] Adonis-Koffy L. (2004). [May Plasmodium falciparum induce a hemolytic uremic syndrome?]. Arch Pediatr.

[180] Noris M., Remuzzi G. (2009). Atypical hemolytic-uremic syndrome. N Engl J Med.

[181] Canpolat C., Pearson P., Jaffe N. (1994). Cisplatin-associated hemolytic uremic syndrome. Cancer.

[182] Boeck S., Geiger S., Schulz C., Heinemann V. (2008). Hemolytic-uremic syndrome associated with gemcitabine treatment for metastatic pancreatic cancer. J Clin Gastroenterol.

[183] Ariyoshi K., Shinohara K., Ruirong X. (1997). Thrombotic thrombocytopenic purpura caused by ticlopidine, successfully treated by plasmapheresis. Am J Hematol.

[184] Andersohn F., Hagmann F.G., Garbe E. (2004). Thrombotic thrombocytopenic purpura/haemolytic uraemic syndrome associated with clopidogrel: report of two new cases. Heart.

[185] Aster R.H. (1993). Quinine sensitivity: a new cause of the hemolytic uremic syndrome. Ann Intern Med.

[186] Gottschall J.L., Neahring B., McFarland J.G., Wu G.G., Weitekamp L.A., Aster R.H. (1994). Quinine-induced immune thrombocytopenia with hemolytic uremic syndrome: clinical and serological findings in nine patients and review of literature. Am J Hematol.

[187] Ubara Y., Hara S., Takedatu H., Katori H., Yamada K., Yoshihara K. (1998). Hemolytic uremic syndrome associated with beta-interferon therapy for chronic hepatitis C. Nephron.

[188] Badid C., McGregor B., Faivre J.M., Guerard A., Juillard L., Fouque D. (2001). Renal thrombotic microangiopathy induced by interferon-alpha. Nephrol Dial Transplant.

[189] Keir L., Moorsel F., Saleem M.A., Richards A. (2012). Beware renal adverse effects of anti-vascular endothelial growth factor treatment. BMJ.

[190] Bonatti H., Brandacher G., Boesmueller C., Cont M., Hengster P., Rosenkranz A.R. (2007). Hemolytic uremic syndrome following Campath-1H induction. Transpl Int.

[191] Abraham K.A., Little M.A., Dorman A.M., Walshe J.J. (2000). Hemolytic-uremic syndrome in association with both cyclosporine and tacrolimus. Transpl Int.

[192] Allan D.S., Thompson C.M., Barr R.M., Clark W.F., Chin-Yee I.H. (2002). Ciprofloxacin-associated hemolytic-uremic syndrome. Ann Pharmacother.

[193] Blumberg A., Studer U., Briner J. (1975). [Hemolytic-uremic syndrome in a young woman following the use of ovulation inhibitors]. Schweiz Med Wochenschr.

[194] Ashouri O.S., Marbury T.C., Fuller T.J., Gaffney E., Grubb W.G., Cade J.R. (1982). Hemolytic uremic syndrome in two postmenopausal women taking a conjugated estrogen preparation. Clin Nephrol.

[195] Au W.Y., Chan K.W., Lam C.C., Young K. (2002). A post-menopausal woman with anuria and uterus bulk: the spectrum of estrogen-induced TTP/HUS. Am J Hematol.

[196] Tumlin J.A., Sands J.M., Someren A. (1990). Hemolytic-uremic syndrome following “crack” cocaine inhalation. Am J Med Sci.

[197] Ardiles L.G., Olavarria F., Elgueta M., Moya P., Mezzano S. (1998). Anticardiolipin antibodies in classic pediatric hemolytic-uremic syndrome: a possible pathogenic role. Nephron.

[198] Barre P., Kaplan B.S., de Chadarevian JP, Drummond KN. (1977). Hemolytic uremic syndrome with hypocomplementemia, serum C3NeF, and glomerular deposits of C3. Arch Pathol Lab Med.

[199] Nesher G., Hanna V.E., Moore T.L., Hersh M., Osborn T.G. (1994). Thrombotic microangiographic hemolytic anemia in systemic lupus erythematosus. Semin Arthritis Rheum.

[200] Hale G.A., Bowman L.C., Rochester RJ, Benaim E, Heslop HE, Krance RA (2005). Hemolytic uremic syndrome after bone marrow transplantation: clinical characteristics and outcome in children. Biol Blood Marrow Transplant.

[201] Lechner K., Obermeier H.L. (2012). Cancer-related microangiopathic hemolytic anemia: clinical and laboratory features in 168 reported cases. Medicine (Baltimore).

[202] Kind T., Levy J., Lee M., Kaicker S., Nicholson J.F., Kane S.A. (2002). Cobalamin C disease presenting as hemolytic-uremic syndrome in the neonatal period. J Pediatr Hematol Oncol.

[203] Lehtinen M.J., Rops A.L., Isenman D.E., van der Vlag J., Jokiranta T.S. (2009). Mutations of factor H impair regulation of surface-bound C3b by three mechanisms in atypical hemolytic uremic syndrome. J Biol Chem.

[204] Morgan H.P., Schmidt C.Q., Guariento M., Blaum B.S., Gillespie D., Herbert A.P. (2011). Structural basis for engagement by complement factor H of C3b on a self surface. Nat Struct Mol Biol.

[205] Jozsi M., Heinen S., Hartmann A., Ostrowicz C.W., Halbich S., Richter H. (2006). Factor H and atypical hemolytic uremic syndrome: mutations in the C-terminus cause structural changes and defective recognition functions. J Am Soc Nephrol.

[206] Jokiranta T.S., Cheng Z.Z., Seeberger H., Jozsi M., Heinen S., Noris M. (2005). Binding of complement factor H to endothelial cells is mediated by the carboxy-terminal glycosaminoglycan binding site. Am J Pathol.

[207] Manuelian T., Hellwage J., Meri S., Caprioli J., Noris M., Heinen S. (2003). Mutations in factor H reduce binding affinity to C3b and heparin and surface attachment to endothelial cells in hemolytic uremic syndrome. J Clin Invest.

[208] Sanchez-Corral P., Gonzalez-Rubio C., Rodriguez de Cordoba S, Lopez-Trascasa M. (2004). Functional analysis in serum from atypical hemolytic uremic syndrome patients reveals impaired protection of host cells associated with mutations in factor H. Mol Immunol.

[209] Sanchez-Corral P., Perez-Caballero D., Huarte O., Simckes A.M., Goicoechea E., Lopez-Trascasa M. (2002). Structural and functional characterization of factor H mutations associated with atypical hemolytic uremic syndrome. Am J Hum Genet.

[210] Morgan H.P., Jiang J., Herbert A.P., Kavanagh D., Uhrin D., Barlow P.N. (2011). Crystallographic determination of the disease-associated T1184R variant of complement regulator factor H. Acta Crystallogr D Biol Crystallogr.

[211] Herbert A.P., Kavanagh D., Johansson C., Morgan H.P., Blaum B.S., Hannan J.P. (2012). Structural and functional characterization of the product of disease-related factor H gene conversion. Biochemistry.

[212] Boyer O., Balzamo E., Charbit M., Biebuyck-Gouge N., Salomon R., Dragon-Durey M.A. (2010). Pulse cyclophosphamide therapy and clinical remission in atypical hemolytic uremic syndrome with anti-complement factor H autoantibodies. Am J Kidney Dis.

[213] Geelen J., van den Dries K., Roos A., van de Kar N, de Kat Angelino C, Klasen I (2007). A missense mutation in factor I (IF) predisposes to atypical haemolytic uraemic syndrome. Pediatr Nephrol.

[214] Chan M.R., Thomas C.P., Torrealba J.R., Djamali A., Fernandez L.A., Nishimura C.J. (2009). Recurrent atypical hemolytic uremic syndrome associated with factor I mutation in a living related renal transplant recipient. Am J Kidney Dis.

[215] Cayci F.S., Cakar N., Hancer V.S., Uncu N., Acar B., Gur G. (2012). Eculizumab therapy in a child with hemolytic uremic syndrome and CFI mutation. Pediatr Nephrol.

[216] Fang C.J., Fremeaux-Bacchi V., Liszewski M.K., Pianetti G., Noris M., Goodship T.H. (2008). Membrane cofactor protein mutations in atypical hemolytic uremic syndrome (aHUS), fatal Stx-HUS, C3 glomerulonephritis, and the HELLP syndrome. Blood.

[217] Noris M., Brioschi S., Caprioli J., Todeschini M., Bresin E., Porrati F. (2003). Familial haemolytic uraemic syndrome and an MCP mutation. Lancet.

[218] Fremeaux-Bacchi V., Sanlaville D., Menouer S., Blouin J., Dragon-Durey M.A., Fischbach M. (2007). Unusual clinical severity of complement membrane cofactor protein-associated hemolytic-uremic syndrome and uniparental isodisomy. Am J Kidney Dis.

[219] Volokhina E., Westra D., Xue X., Gros P., van de Kar N, van den Heuvel L. (2012). Novel C3 mutation p.Lys65Gln in aHUS affects complement factor H binding. Pediatr Nephrol.

[220] Malina M., Gulati A., Bagga A., Majid M.A., Simkova E., Schaefer F. (2013). Peripheral gangrene in children with atypical hemolytic uremic syndrome. Pediatrics.

[221] Ozel A., Caliskan U., Gucer S. (2003). Peripheral gangrene complicating hemolytic uremic syndrome in a child. Pediatr Nephrol.

[222] Kaplan B.S., Garcia C.D., Chesney R.W., Segar W.E., Giugno K., Chem R. (2000). Peripheral gangrene complicating idiopathic and recessive hemolytic uremic syndromes. Pediatr Nephrol.

[223] Loirat C., Macher M.A., Elmaleh-Berges M., Kwon T., Deschenes G., Goodship T.H. (2010). Non-atheromatous arterial stenoses in atypical haemolytic uraemic syndrome associated with complement dysregulation. Nephrol Dial Transplant.

[224] Vergouwen M.D., Adriani K.S., Roos Y.B., Groothoff J.W., Majoie C.B. (2008). Proximal cerebral artery stenosis in a patient with hemolytic uremic syndrome. AJNR Am J Neuroradiol.

[225] Sallee M., Daniel L., Piercecchi M.D., Jaubert D., Fremeaux-Bacchi V., Berland Y (2010). Myocardial infarction is a complication of factor H-associated atypical HUS. Nephrol Dial Transplant.

[226] Larakeb A., Leroy S., Fremeaux-Bacchi V., Montchilova M., Pelosse B., Dunand O. (2007). Ocular involvement in hemolytic uremic syndrome due to factor H deficiency--are there therapeutic consequences?. Pediatr Nephrol.

